# Virtual reality as a metacognitive training tool for lucid dreaming

**DOI:** 10.3389/fpsyg.2026.1924370

**Published:** 2026-09-28

**Authors:** Andrea Signorelli, Francesco Tormen, Bruna Dal Bianco, Nicola De Pisapia

**Affiliations:** 1 Consciousness, Creativity and Cognitive Control Lab, Department of Psychology and Cognitive Science, University of Trento, Rovereto, Italy; 2 Fondazione Bruno Kessler, Trento, Italy; 3 Italian Buddhist Union Research Center, Rome, Italy

**Keywords:** cognitive training, dream consciousness, lucid dreaming, lucid dreaming induction, metacognition, open-monitoring meditation, reality monitoring, virtual reality

## Abstract

**Introduction:**

Lucid dreaming (LD) involves awareness of being in a dream during REM sleep and depends on the reactivation of metacognitive and reality-monitoring processes. This study tested whether a virtual reality (VR) intervention designed to rehearse lucid-dream-relevant metacognitive routines increases LD rates beyond baseline training, and whether generic VR exposure produces similar effects.

**Materials and methods:**

Thirty-eight participants with low previous LD experience completed a 5-week program of daily dream diaries and reality checks, assigned to: VR-experimental (*n* = 13), standard training plus eight LucidDreamingVR sessions preceded by brief open monitoring (OM) meditation; VR-control (*n* = 12), standard training plus eight LD-unrelated VR sessions; or Control (*n* = 13), standard training only. Dreams were classified as ordinary, semi-lucid, or lucid; semi-lucid and lucid dreams were treated as lucid-like.

**Results:**

VR-experimental participants reported the highest number of lucid like dreams (26), followed by Control (11) and VR-control (4). A Poisson model with total recalled dreams as offset showed that lucid-like dream rates were significantly higher in VR-experimental than in VR-control, RR = 8.60, 95% CI [3.00, 24.64], p_Holm <0.001, and Control, RR = 4.00, 95% CI [1.97, 8.09], p_Holm <0.001. Responder rates showed a convergent pattern, with at least one lucid-like dream reported by 84.6% of VR-experimental participants, 61.5% of Control participants, and 33.3% of VR-control participants, showing that VR exposure alone was insufficient.

**Discussion/conclusion:**

Dream-oriented VR training preceded by OM meditation may facilitate LD by scaffolding metacognitive monitoring from wakefulness to REM sleep.

## 1 Introduction

Lucid dreaming (LD) is the experience of becoming aware that one is dreaming while the dream is ongoing, often with some degree of intentional influence over the dream scenario (De Pisapia, 2021; Tormen, 2024; Voss et al., 2009). LD is of interest because it combines core features of REM dreaming, such as perceptual immersion and narrative absorption, with waking-like capacities such as self-monitoring, volition, and metacognitive insight (Voss et al., 2009). From a cognitive-enhancement perspective, LD is therefore relevant as a form of state-awareness, in which metacognitive monitoring and cognitive control are recruited within an immersive experiential context.

Neurobiological evidence supports this hybrid characterization. Lucid REM has been associated with altered oscillatory dynamics and partial re-engagement of prefrontal–parietal regions implicated in metacognition and cognitive control (Denzer et al., 2024; Dresler et al., 2012; Voss et al., 2009). Trait-level findings in frequent lucid dreamers further suggest links between lucidity and frontoparietal systems supporting metacognitive monitoring (De Pisapia et al., 2026; Filevich et al., 2015; Voss et al., 2009). At the phenomenological level, lucidity can introduce an observer-like stance toward ongoing experience and a degree of experiential detachment. Notably, these qualities also figure prominently in contemplative descriptions of cultivated awareness (Baird et al., 2019; De Pisapia, 2021). This linkage is more than conceptual: metacognitive and attentional training in wakefulness has been proposed as one pathway by which lucidity may become more likely to arise in sleep (Baird et al., 2019; Voss et al., 2009).

Although about half of individuals report having experienced at least one lucid dream, regular LD is considerably less common (Saunders et al., 2016). A range of approaches has therefore been developed to facilitate lucidity, including cognitive and behavioral techniques, sleep-related external stimulation, pharmacological interventions, and cortical stimulation (Stumbrys et al., 2012; Tan and Fan, 2023). However, empirical support varies substantially across methods. A recent systematic review identified mnemonic induction of lucid dreams (MILD) as the best-supported cognitive induction technique, whereas evidence for several other approaches, including reality checks (RCs) when evaluated independently, remained limited or ambiguous (Tan and Fan, 2023). Despite this limited evidence, RCs are theoretically relevant because they can be understood as a simple cognitive-training procedure targeting reality monitoring, prospective memory, and reflective evaluation (Dyck et al., 2017; Stumbrys et al., 2012). They typically involve repeatedly asking whether one is dreaming, followed by a behavioral test expected to differ between waking and dreaming (Dyck et al., 2017).

Through repetition, RCs are intended to become habitual and to transfer into dreams via “dream residue,” where they may be triggered by dream signs (DSs) that are unusual, surprising, or incongruent events, and thereby precipitate insight into the dream state (Stumbrys et al., 2012). In practice, RCs may also be cued by learned associations, such as responding to a notification or auditory prompt that has been linked to reflective questioning (Schmid and Erlacher, 2020). More generally, RC-based training attempts to operationalize a core requirement of lucidity: interrupting immersion with a brief act of epistemic evaluation while remaining engaged with ongoing experience. In cognitive-enhancement terms, the target process is the flexible deployment of metacognitive monitoring in response to contextual anomalies.

Meditation, particularly open-monitoring (OM) practice, offers another route to training meta-awareness. Whereas, focused-attention meditation emphasizes sustaining attention on a specific object, OM involves non-reactive monitoring of the contents of experience as they unfold from moment to moment (Lutz et al., 2008). OM may therefore support a domain-general state of receptive metacognitive monitoring, in which attention remains sensitive to changes in ongoing experience without being organized around predetermined contents or becoming fully absorbed in them. In Tibetan contemplative traditions, OM meditation and LD are often framed as complementary daytime and nighttime practices (Tormen, 2024; Wallace, 2010). OM cultivates non-reactive awareness of ongoing mental events, and recent evidence links OM-related practice profiles with higher lucid dream frequency and trait meta-awareness (Gerhardt and Baird, 2024).

A central obstacle for novice lucid dreamers is that RC-based induction depends on recognizing anomalies and responding to them with reflective questioning, yet waking life rarely provides experiences that are sufficiently “dream-like” to make the need for reality monitoring feel immediate and compelling (Stumbrys et al., 2012). As a result, RC practice can become mechanical, and its transfer to dreams may be unreliable. This limitation motivates interventions that can provide repeated, embodied exposure to salient prediction-violations, which are events that feel implausible within ordinary waking assumptions, and that can therefore strengthen the coupling between anomaly detection and metacognitive checking.

Virtual reality (VR) is a promising medium for this purpose and, more broadly, for process-specific cognitive training. VR-based interventions can reproduce complex, ecologically engaging scenarios while retaining experimental control and safety, and across applied domains VR training has been shown to support both performance and cognitive skill acquisition (Cariati et al., 2025; Mitsea et al., 2023). Beyond technical training, VR has been used to support metacognitive and affective skills, including mindfulness-oriented interventions and guided meditation (Jo et al., 2024; Ma et al., 2023). Relatedly, immersive manipulations can modulate cognitive flexibility (Rastelli et al., 2022), a capacity relevant to reappraising ongoing experience when ordinary assumptions are violated.

These general affordances become specifically pertinent for LD because VR can deliberately construct perceptual and narrative conditions that resemble dream phenomenology (unusual events, violated physical constraints, and rapid contextual shifts), while the user remains awake and capable of reflective learning. Dream-like qualities of VR have also begun to be formalized in design-oriented work, including proposed guidelines for creating immersive experiences that parallel aspects of LD (Kitson et al., 2022). Moreover, empirical studies at the intersection of immersion and altered experience further motivate VR as a controlled platform for simulating dream-like bizarreness (Denzer et al., 2024). At the same time, spontaneous dream incorporation of videogame elements (Gackenbach et al., 2011) may provide a mechanism through which metacognitive skills rehearsed during wakefulness are transferred to the dream state.

In a controlled experiment, Gott et al. (2021) found that VR-enhanced lucid dream training increased dream lucidity relative to comparison conditions. This suggests that VR may augment induction practices by providing a metacognitive rehearsal space in which users experience, rather than merely imagine, the transition from taking an environment for granted to questioning its reality.

Building on these premises, LucidDreamingVR was developed as a multi-component training intervention combining brief OM-based preparation with progressive interactive VR training in DS recognition, RC execution, stabilization, and volitional control. The two components were intended to serve complementary functions: OM was included to establish a receptive and non-reactive mode of monitoring, whereas the interactive VR component directed this general orientation toward LD-specific contents and provided embodied opportunities to rehearse them. The pedagogical goal of the LucidDreamingVR package is to make anomaly detection and reflective questioning experientially salient by allowing novices to encounter controlled impossibilities in VR.

The present study tested whether 1 month of LucidDreamingVR, combined with RC-based baseline training, increases lucid-like dreaming relative to baseline training alone. To distinguish dream-oriented VR content from VR exposure *per se*, a VR-control condition using a visually similar but LD-unrelated application was included, alongside a no-VR baseline condition. This comparison makes it possible to assess whether any observed effects reflected the specific metacognitive content of the LD-oriented intervention, rather than non-specific factors associated with VR exposure, novelty, or engagement.

State-level effects of VR were assessed with the Phenomenology of Consciousness Inventory (Pekala, 1991). Lucidity outcomes were tracked daily and quantified both by participants' direct classification of lucid and prelucid dreams (treated as a continuum of “lucid-like” experiences) and by validated phenomenological dimensions of dream consciousness using the LuCiD scale (Baird et al., 2018; Mallett et al., 2021; Tyson et al., 1984; Voss et al., 2013). By testing whether immersive, dream-oriented metacognitive training produces gains beyond both baseline LD practice and non-specific VR exposure, the study contributes to cognitive-enhancement research on how reflective awareness can be trained in highly immersive experiential contexts.

## 2 Materials and methods

### 2.1 Study overview

This study used a three-group controlled design to test whether LD-specific VR training increases dream lucidity beyond baseline LD training and non-specific VR exposure. All participants followed a standard LD protocol based on daily RCs and dream diary maintenance. Participants were assigned to one of three conditions: VR-Experimental, using LucidDreamingVR; VR-Control, using a visually similar LD-unrelated VR application; or Control, with no VR exposure.

A schematic overview of the full experimental protocol is provided in Figure 1.

**Figure 1:**
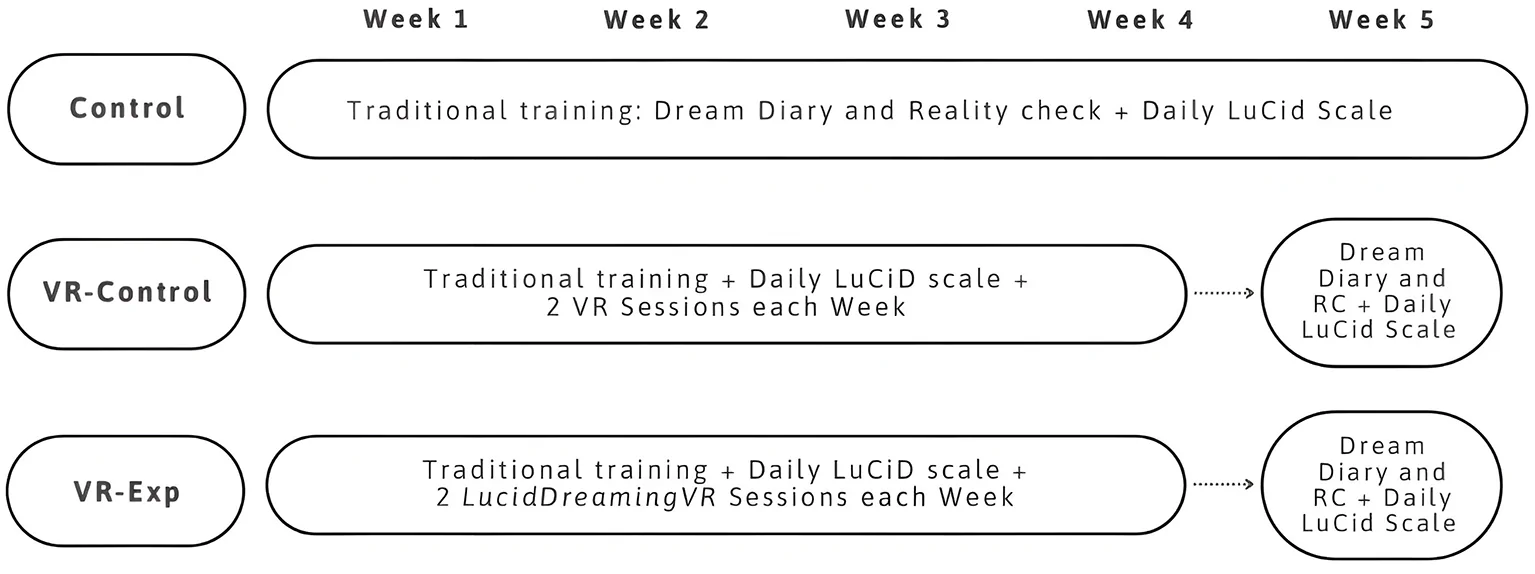
Study design and timeline. Schematic overview of the 5-week experimental protocol across the three groups. All participants followed a baseline LD training consisting of daily RCs, dream diary maintenance, and daily completion of the Lucidity and Consciousness in Dreams (LuCiD) scale. The Control group completed the baseline training for all 5 weeks. The VR-Control group completed the baseline training plus 2 weekly laboratory sessions using a content-neutral VR application during Weeks 1–4, followed by baseline training only in Week 5. The VR-Experimental (VR-Exp) group completed the baseline training plus 2 weekly laboratory sessions using LucidDreamingVR (completed of a OM preliminary meditation and the actual LD training) during Weeks 1–4, followed by baseline training only in Week 5.

At study entry, participants attended an in-person laboratory session for consent, procedural instructions, baseline-training instruction, and motivation assessment. Participants assigned to the Control group were then dismissed and began the at-home training immediately, whereas participants assigned to the VR-Exp and VR-Control groups completed their first VR session during this initial laboratory visit.

The VR intervention comprised eight laboratory sessions over 4 weeks, delivered twice weekly. In the VR-Exp group, each LucidDreamingVR session included a seated guided OM meditation (~15 min) followed by a room-scale interactive component (~20 min) targeting dream-sign recognition, RC execution, and, in later sessions, lucid-dream–specific goal-directed actions.

Participants in the VR-Control group completed eight sessions (20 mins each) with a visually similar, lucid-dream–unrelated VR application using room-scale tracking while standing; the task consisted of freely exploring the environment and locating hidden baseballs, and no meditation component was included.

Thus, the active interactive component lasted approximately 20 mins in both VR conditions.

The Phenomenology of Consciousness Inventory (Pekala, 1991) questionnaire was administered after the first and the last VR session. Throughout the study, participants completed a daily questionnaire containing the Lucidity and Consciousness in Dreams scale (Voss et al., 2013) items. After 4 weeks, all participants completed a final fifth week during which only the baseline training and daily questionnaires continued, for a total study duration of 5 weeks (35 consecutive days).

Both VR applications were accessed using a Meta Quest 3 headset connected via Air Link to a Victus TG02 gaming desktop equipped with an Intel Core i7-14700F processor, 32 GB RAM, and an NVIDIA GeForce RTX 4060 GPU.

### 2.2 Participants

A total of 38 participants were recruited through the SONA platform at the University of Trento or through posters hung in the University's public spaces. Inclusion criteria required less than 2 h of prior VR exposure, no previous lucid dream training, and a spontaneous lucid dream recall of maximum 3–5 dreams per year. Additional eligibility requirements included being between the ages of 18 and 40 and reporting no diagnosed neurological or psychiatric conditions. Gender was self-reported. Sex assigned at birth was not collected. The sample size was determined with reference to previous research using a comparable design (Gott et al., 2021), while also taking into account the practical and resource constraints associated with data collection.

Participants who passed the demographic screening and consented to participate were assigned to one of three groups using stratified randomization, balancing gender, dream recall and lucid dream recall. The final sample consisted of *N*_tot_ = 38 participants (*M*_age_ = 23.1 years, SD_age_ = 3.85; 21 female, 16 male, 1 non-binary/other), divided into VR-Exp (*N*_E_ = 13), VR-Control (*N*_CVR_=12), and Control (*N*_C_ = 13) groups.

All participants completed the full 5-week protocol.

### 2.3 Measures

#### 2.3.1 Demographic survey

A demographic screening questionnaire was administered to assess eligibility criteria. It included items on age, gender, education, spoken languages, contact information, smartphone model (used to provide a correct version of the companion app), and instructions to create a personal identification code, that would then be used to anonymize their responses during data processing. Participants reported their prior experience with VR using a six-points scale that captured the approximate ranges of hours of use (0 = never tried, 1 = 1 h or less, 2 = between 1 and 2 h, 3 = between 2 and 5 h, 4 = between 5 and 10 h, and 5 = 10 h or more). General dream recall frequency and lucid dream recall frequency were assessed using a 14-point scale (Baird et al., 2018). An example of lucid dream and an example of an ordinary dream were presented to ensure participants' comprehension.

#### 2.3.2 Daily questionnaire

A daily self-report questionnaire was used to monitor participants' dream recall and dream lucidity throughout the study period. When at least one dream was reported, participants completed the full LuCiD scale.

Lucidity was assessed through a direct question (“Have you had a lucid dream tonight?”), with three possible responses: yes, no, and maybe. Responses marked as maybe were classified as semi-lucid dreams, broadly corresponding to intermediate levels of dream awareness described by Mallett et al., 2021.

This operational choice was further supported by a *post-hoc* phenomenological inspection of the available *maybe* reports. The narratives were found to span a lucid-awareness continuum, ranging from pre-lucid forms of anomaly detection or dream-state questioning to partial, unstable, or explicitly lucid experiences. Importantly, none of the available reports described sleep paralysis, clear sleep-related out-of-body experiences, or other phenomena that would indicate that the *maybe* category primarily reflected states unrelated to dream lucidity. External coding was used descriptively to characterize the phenomenological range of the category rather than to replace participants' original self-classifications. Full reports of “maybe” lucid dreams are available, along with their qualitative classification, in the Supplementary materials. For primary analyses, lucid and semi-lucid dreams were combined as “lucid-like” dreams, consistent with a graded view of LD and allowing detection of partial or unstable forms of lucidity (Mallett et al., 2021).

When lucidity was reported, participants were asked to provide a written dream narrative; narratives were not requested for ordinary dreams. The questionnaire also recorded the number of RCs performed the previous day.

#### 2.3.3 Lucidity and consciousness in dreams scale

Dream consciousness was assessed using the Lucidity and Consciousness in Dreams scale (LuCiD scale) (Voss et al., 2013), whose eight subscales capture primary and secondary aspects of consciousness and discriminate between lucid and non-lucid dreams. The LuCiD scale was originally developed to assess the level of consciousness activation within a single dream.

As this study's premises support the conception of lucidity as a graded continuum, assessing cognitive activation at the level of the entire night was deemed preferable to considering only single experiences. This need was counterbalanced with the high workload requested in order to participate in the study, leading to the solution of asking participants to complete the LuCiD scale only once per night considering all dreams remembered from that night, instead of asking them to complete it once for every single dream.

Specifically, for each item participants were instructed to report the highest rating that applied to at least one of the dreams recalled. For example, a participant who reported three dreams in the same night of which only one was lucid would still select a high score on the item “While dreaming, I was aware of the fact that the things I was experiencing in the dream were not real.”

This approach also made it possible to capture features occurring in ordinary dreams that might otherwise have gone unrecorded, thereby preserving potentially important information about the overall effect of the training in modulating dimensions related to dream lucidity.

#### 2.3.4 Phenomenology of consciousness inventory

The Phenomenology of Consciousness Inventory (PCI) is a self-report questionnaire designed to quantify subjective features of conscious experience across multiple phenomenological dimensions, including perceptual, cognitive, affective, attentional, and metacognitive aspects of experience. It was used to assess if and how the two VR applications differentially affected the state of consciousness of the participants.

### 2.4 Baseline training

Baseline LD training targeted dream recall and habitual RC practice.

To improve dream recall, participants kept a dream diary every morning immediately after waking, including days without remembered dreams (Schredl, 2002).

Participants were also instructed to perform RCs approximately 10 times per day, especially when encountering something strange or spontaneously thinking of it. A set of 12 alarms was also installed on participants' phones and scheduled to ring hourly, between 9 a.m. and 9 p.m., every day for the duration of the experiment; their ringing (or notification viewing) was associated with performing a RC.

Participants received a curated list of commonly used RCs (LaBerge and Rheingold, 1991), including nose-pinching and attempting to breathe, inspecting the hands for anomalies, and flipping a light switch to check for expected feedback.

They were encouraged to adopt the RCs they found most reliable or contextually appropriate, while maintaining the same overall training rationale across groups.

### 2.5 Virtual reality applications

#### 2.5.1 LucidDreamingVR

LucidDreamingVR is a VR-based application designed to deliver a secularized, widely accessible lucid-dream training inspired by Tibetan dream yoga practices. The project was commissioned and overseen by the UBI Research Center.

The application implements a progressive protocol of eight VR sessions (~35 mins each), delivered twice weekly over 4 weeks. Each session begins with a ~15-min guided OM meditation in a naturalistic immersive environment, inspired by contemplative practices associated with Tibetan dream yoga and the work of Alan Wallace (Wallace, 2010). Across sessions, the meditation gradually shifts from bodily sensations and breathing to monitoring thoughts and emotions.

This OM component was designed as a preparation for the interactive training rather than as a stand-alone LD induction technique. Its exercises concerned the open and non-reactive monitoring of bodily sensations, breathing, thoughts, and emotions and did not introduce any LD-specific instruction. OM was intended to establish a general experiential and attentional context in which participants could engage with the LD-specific training, rather than to provide that training itself. Consistent with this role, OM was limited to the eight laboratory sessions and was not assigned as a daily at-home practice.

The second half of each session (approximately 20 mins) requires active engagement. Guided by a voice-over, participants are invited to stand and explore an environment that reproduces characteristic features of dreaming, including incongruous or out-of-place objects, details that change when attention is redirected, and narrative events that are surprising, ambiguous, or mysterious.

Through sessions, participants are progressively trained in three classes of operations relevant to the recognition, maintenance, and intentional exploration of LD. First, they practice detecting DSs, learning to use them as cues for reflective awareness. Second, they rehearse RCs such as checking visual details, inspecting their hands, and testing gravity.

From session three onward, successful DS and RC tasks trigger a simulated transition into lucidity through changes in the environment's visual properties (Shashkov et al., 2023). Lucidity is treated as reversible and must be maintained over time through continued DS detection and RC execution, approximating deepening techniques common in lucid dream practice (Shashkov et al., 2026).

In the final sessions, participants rehearse lucid-dream–specific abilities, or “superpowers,” such as high jumps, flying, and changing body size through verbal intention. Adapted from Tibetan dream yoga practices (Tormen, 2024), these exercises are used here to support goal-directed rehearsal in lucid-dream contexts.

This rationale is consistent with accounts linking lucid-dream frequency to prospective memory, which is central to RC practice and mnemonic induction methods such as MILD (LaBerge and Rheingold, 1991).

Repeated first-person enactment in VR may also support procedural learning of lucid-dream action sequences, such as detecting anomalies, performing RCs, maintaining reflection, and pursuing lucid goals. This extends to dream training, a logic already used in VR-based procedural training in other domains (Abbas et al., 2023).

Across sessions, interaction mechanics, environments, and characters are retained and gradually elaborated (representative screenshots are shown in Figure 2). The first four sessions function mainly as tutorials in a realistic domestic environment, whereas the final four introduce more narrative, dream-like scenarios and the superpowers described above.

**Figure 2:**
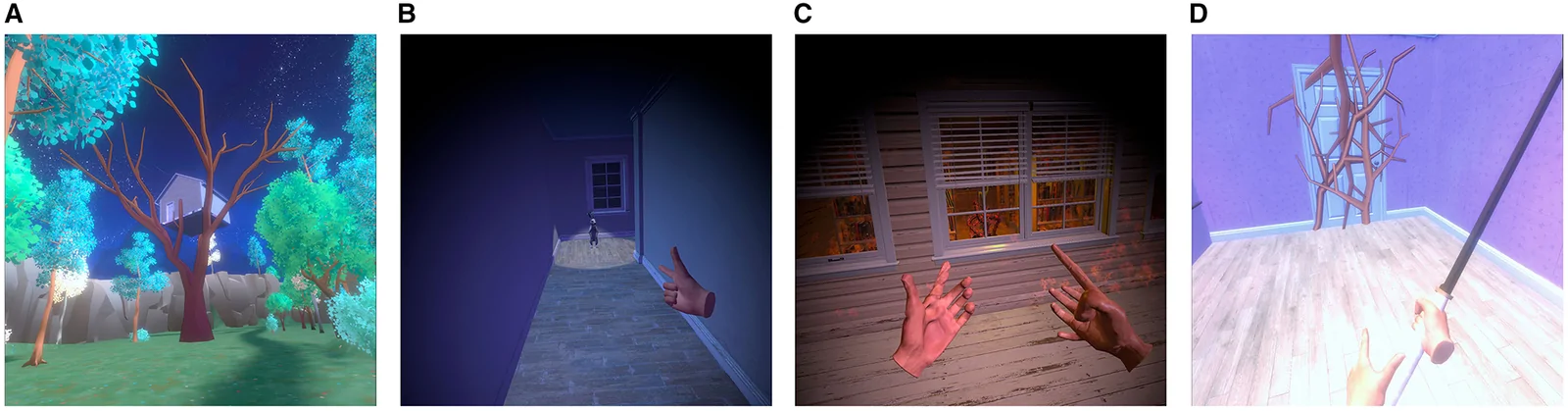
Screenshots of LucidDreamingVR. **(A)** Represents one visual of the Grove environment, as found in the eighth session, and shows the dream-like nature of the scenes found throughout the training. **(B)** Shows a strange situation that should be recognized as potential DS. **(C)** Shows the act of doing a RC. In **(D)** the user is currently “lucid,” as evidenced by the brightness of the image, and has just made the sword appear in order to cut the branches that block the door.

#### 2.5.2 Control VR application

The VR-Control group experienced a VR application consisting of eight 20-min sessions. While the visual assets and environments largely overlapped with those of LucidDreamingVR, the content was unrelated to LD or dream training. The application also included no narrative structure. Users navigated the environment through tracked body movements and Meta Quest controllers, with no graphical user interface elements.

Participants were instructed to explore while locating baseballs hidden throughout the scene. No performance feedback was provided. The task was framed as a relaxed, curiosity-driven exploration, with the baseballs serving solely as markers of attentional engagement. Figure 3 shows some screenshots of the control application, with the target baseballs highlighted in a green circle.

**Figure 3:**
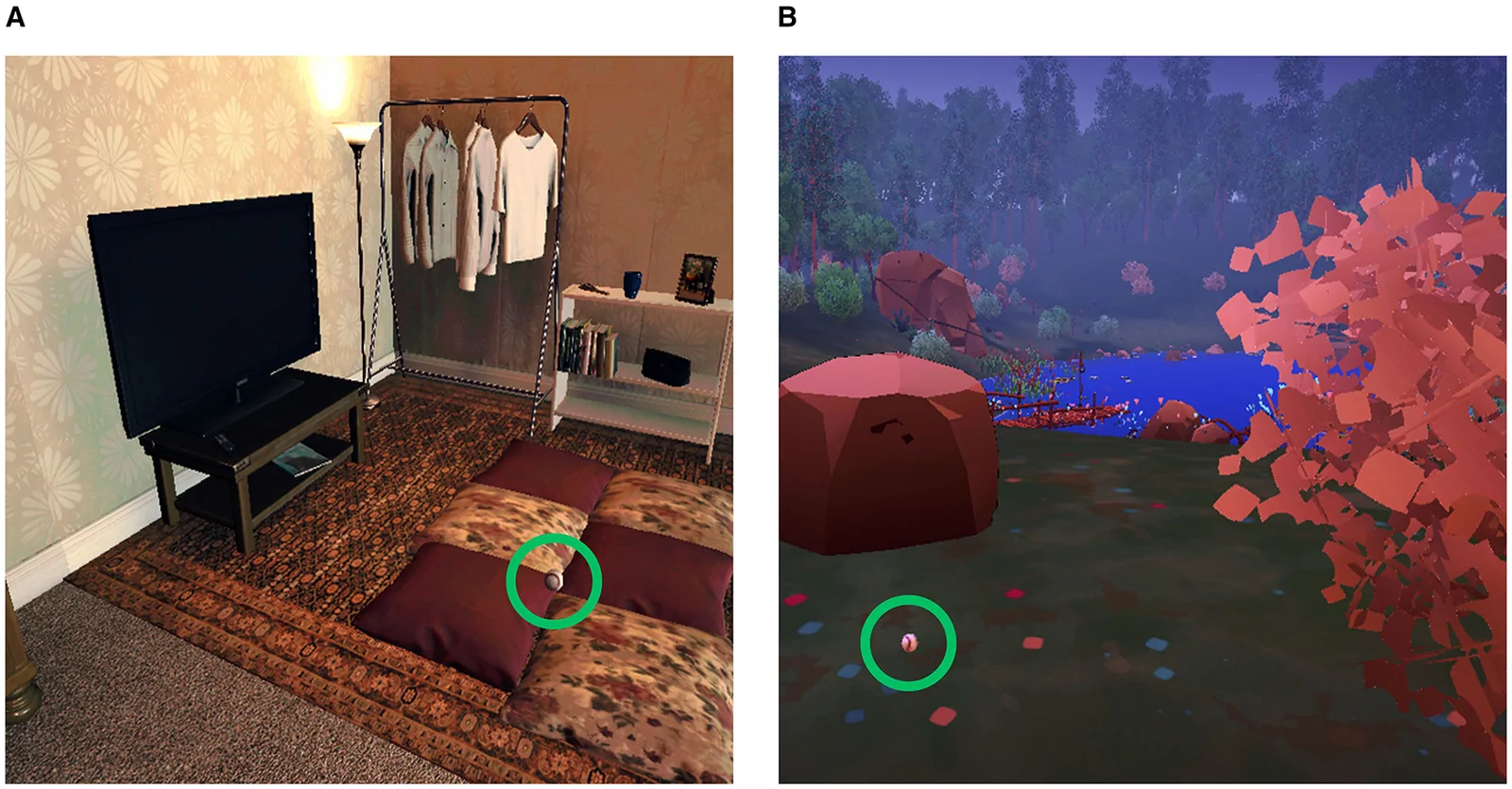
Screenshots of the VR application used by Control-VR participants. Environments proposed during the sessions were alternatively indoor **(A)** or outdoor **(B)** and were similar to those contained in LucidDreamingVR but devoid of dream-like elements or LD-oriented exercises. Instead, baseballs (highlighted in the figure by green rings) were spread around the environments, and finding them was proposed as an attentive task.

### 2.6 Ethical approval and consent

Participants received an initial description of the study structure, duration, and requirements through a demographic screening questionnaire.

No detailed medical records were collected; eligibility included self-reported absence of diagnosed neurological or psychiatric conditions. Recorded data were limited to non-identifiable demographic variables and non-sensitive self-reports regarding prior VR exposure and dream-related experience.

During an introductory session, participants received a detailed explanation of all procedures and provided written informed consent, including consent for data processing and privacy protection.

All data were pseudonymized using participant-generated identification codes and stored on repositories compliant with current data protection and privacy regulations. The study was conducted in accordance with the principles of the Declaration of Helsinki and was approved by the University of Trento Ethical Committee (protocol code 2024-062).

### 2.7 Data manipulation and statistical analysis

#### 2.7.1 Pre-processing and database structure

All data processing and analyses were conducted using Jupyter Notebook and Python libraries. Questionnaire data were exported from Qualtrics and processed on a secure local computer. No data were uploaded to external servers or third-party platforms during analysis.

In the final dataset, each row corresponded to a single daily entry and was linked to the participant's pseudonymized identification code. The complete dataset comprised 1,041 rows and 90 columns, revealing a mean number of 27.4 entries of the daily questionnaire per participant (SD = 7.09).

#### 2.7.2 Assessment of demographic differences across groups

Demographic measures obtained through the initial survey were compared across groups, to assess potential significant differences that may impact the final results of the research.

Depending on normality and variance of the distributions, one-way ANOVA or Mann–Whitney *U* were applied. The same tests were used to check differences between participants that reported at least one lucid or semi-lucid dream during the experiment and those who didn't.

#### 2.7.3 Lucid dreaming frequency assessment

The primary outcome used to assess training effects was the number of lucid-like dreams reported during the experiment. For each participant, this was quantified by counting dream narratives classified as lucid or semi-lucid. These values were summed within each group and visualized on a weekly basis. In addition to between-group comparisons, within-group distributions of lucid and semi-lucid dreams were examined to capture individual differences in achieving lucidity.

To compare the rate of lucid-like dreams across experimental groups, analyses were conducted at the participant level to account for the fact that each participant could contribute multiple diary entries. Entries with zero recalled dreams were excluded. For each participant, the number of lucid-like dreams was summed across valid diary entries, and this count was modeled as a function of experimental group using a Poisson regression with a log link. The logarithm of the total number of recalled dreams was included as an offset, allowing the model to compare the rate of lucid-like dreams per recalled dream across groups. Overdispersion was assessed using the Pearson chi-square statistic divided by the residual degrees of freedom. Pairwise group contrasts were corrected using the Holm-Bonferroni method. A negative binomial model with the same structure was also estimated as a sensitivity analysis.

A responder analysis was conducted to examine whether the proportion of participants who experienced at least one lucid-like dream differed across groups. Participants were classified as responders if they reported at least one lucid-like dream across valid diary entries. Given the small group sizes and the categorical nature of the outcome, pairwise group comparisons were performed using Fisher's exact tests. Odds ratios, 95% confidence intervals, and Holm–Bonferroni-adjusted *p*-values were reported.

For the VR-Exp group, a qualitative evaluation of reported lucid-like dream descriptions was performed to look for possible LucidDreamingVR content incorporation and potential effects on lucidity.

Dream recall was expected to increase relative to baseline due to systematic dream reporting (Schredl and Erlacher, 2011). Accordingly, the number of recalled dreams per week was averaged within each group and compared to baseline values derived from the demographic questionnaire.

#### 2.7.4 LuCiD scale analysis

The internal reliability of the LuCiD scale was assessed using Cronbach's α, that provided an index of the extent to which the LuCiD subscales could be considered reliable measures of the underlying cognitive characteristics of dream mentation (Henson, 2001).

The LuCiD Scale subdimensions were used to examine differences in the cognitive characteristics of nocturnal mentation across ordinary, semi-lucid, and lucid dreams. Nights without recalled dreams were excluded, and weekly subscale values were averaged per group and visualized using matplotlib and seaborn.semi-lucidTo address the non-independence of repeated dream reports, all analyses were conducted at the subject level by computing mean scores separately for each dream type, yielding one aggregated value per condition per participant.

Differences were assessed using pairwise Mann–Whitney *U* tests. To account for multiple testing across the three pairwise contrasts, *p*-values were adjusted using the Holm–Bonferroni correction. Although dream categories partly overlapped within individuals, they were treated as independent for statistical purposes due to the small and unbalanced samples and the need to prioritize robustness over model complexity.

#### 2.7.5 PCI analysis

Statistical tests were conducted on PCI subscales to evaluate (i) changes in the quality of consciousness alteration between the first and last sessions of LucidDreamingVR and the VR-Control app (within-group analyses), and (ii) differences between the two applications at the first and last sessions (between-group analyses).

Given the small sample size (13 and 12 entries per session), only non-parametric tests were used. Wilcoxon signed-rank tests were applied for within-group comparisons, while Mann–Whitney *U* tests were used for between-group comparisons at each session. Holm–Bonferroni correction was applied separately to the two sets of tests.

Since only one comparison survived Holm–Bonferroni correction, the analysis should be considered qualitative, and only contrasts reaching significance at least before correction are reported in the main text. The whole results table is available in the Supplementary materials.

## 3 Results

### 3.1 Assessment of demographic measures

Groups did not differ significantly on demographic variables. However, baseline dream recall and baseline lucid dream recall were numerically highest in the Control group. Participants who reported at least one lucid-like dream during the study did not differ significantly from those who did not on any demographic variable, including self-reported gender. Refer to Table 1 for full participants characteristics.

**Table 1:** Descriptive statistics of participants across groups (Mean ± SD).

Variable	All (*N* = 38)	Control (*N* = 13)	VR-Control (*N* = 12)	VR-Exp (*N* = 13)
Females (count)	21	7	5	9
Age (years)	23.11 ± 3.85	22.46 ± 3.50	23.50 ± 4.01	23.38 ± 4.23
Dream recall	10.55 ± 3.10	11.77 ± 3.42	10.00 ± 2.70	9.85 ± 2.97
Lucid dream recall	1.18 ± 1.57	1.54 ± 1.76	0.75 ± 1.22	1.23 ± 1.69
VR experience (hours)	0.89 ± 0.86	0.84 ± 0.80	1.00 ± 0.74	0.85 ± 1.06

### 3.2 Lucid dreaming frequency assessment

#### 3.2.1 Overall lucid-like dreams distribution

Table 2 summarizes the distribution of lucid-like dream production across groups. At the participant level, at least one lucid-like dream was reported by 11/13 participants in the VR-Exp group (84.6%), compared with 8/13 participants in the Control group (61.5%) and 4/12 participants in the VR-Control group (33.3%). Thus, the proportion of participants who experienced at least one lucid-like dream was highest in the VR-Exp group, intermediate in the Control group, and lowest in the VR-Control group. This responder measure captures how widely lucid-like dreaming occurred within each condition, rather than being driven only by repeated reports from a small number of participants.

**Table 2:** Training-related production of dreams by group (counts).

Outcome/measure	All	Control	VR-Control	VR-Exp
Dream counts				
All dreams	873	367	288	218
Lucid dreams	12	4	1	7
Semi-lucid dreams	29	7	3	19
Total lucid-like dreams	41	11	4	26
Ordinary dreams	832	356	284	192
Participant outcomes, n (%)				
Participants	38	13	12	13
Had at least one lucid dream	10 (26.3%)	3 (23.1%)	1 (8.3%)	6 (46.2%)
Had at least one semi-lucid dream	17 (44.7%)	5 (38.5%)	3 (25%)	9 (69.2%)
Had at least one lucid-like dream	23 (60.5%)	8 (61.5%)	4 (33.3%)	11 (84.6%)
Lucid-like dreams per participant, mean ±SD				
Only responsive participants	1.78 ± 1.44	1.38 ± 0.52	1.00 ± 0.00	2.36 ± 1.91
Including all participants	1.08 ± 1.42	0.85 ± 0.80	0.33 ± 0.49	2.00 ± 1.96

The number of ordinary dreams was obtained by subtracting the number of reported lucid and semi-lucid dreams from the total number of dreams.

To account for differences in overall dream recall, lucid-like dream counts were also analyzed as rates relative to the total number of recalled dreams. Descriptively, lucid-like dreams represented 11.82% of recalled dreams in the VR-Exp group, compared with 2.96% in the Control group and 1.37% in the VR-Control group. A Poisson regression model with the logarithm of total recalled dreams entered as an offset indicated that the VR-Exp group had a significantly higher rate of lucid-like dreams than both the VR-Control group, RR = 8.60, 95% CI [3.00, 24.64], and *p*_Holm <0.001, and the Control group, RR = 4.00, 95% CI [1.97, 8.09], and *p*_Holm <0.001. The Control and VR-Control groups did not differ significantly, RR = 2.15, 95% CI [0.68, 6.76], and *p*_Holm = 0.190. There was no evidence of overdispersion in the Poisson model, Pearson chi-square/df = 0.848, and a negative binomial sensitivity analysis yielded the same pattern of results, supporting the robustness of these group differences.

The responder analysis provided convergent, although more conservative, evidence. Pairwise Fisher's exact tests showed that participants in the VR-Exp group had significantly higher odds of reporting at least one lucid-like dream than participants in the VR-Control group, 84.6% vs. 33.3%, OR = 10.99, 95% CI [1.60, 76.92], and *p*_Holm = 0.046. No other responder comparison was significant after Holm–Bonferroni correction. Together, the rate-based and responder analyses suggest that the VR-Exp condition was associated not only with a higher frequency of lucid-like dreams among recalled dreams, but also with a broader distribution of lucid-like experiences across participants.

The same descriptive pattern was observed in the event counts shown in Figure 4. The VR-Exp group accounted for the largest number of lucid-like dreams, followed by the Control group, whereas the VR-Control group reported the fewest. Importantly, this pattern did not appear to reflect greater overall dream recall in the VR-Exp group, which reported the lowest number of ordinary dreams across the study period.

**Figure 4:**
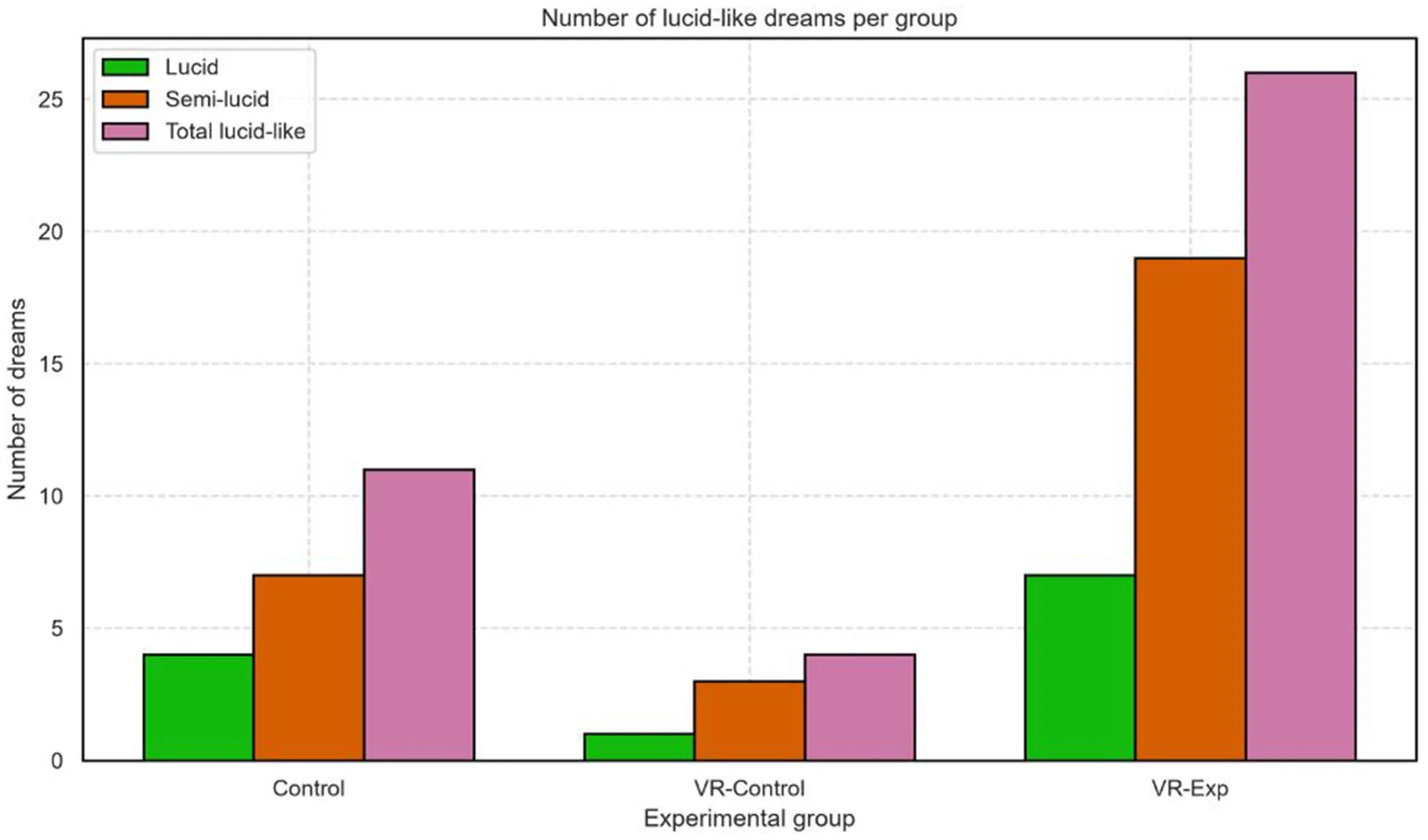
Total number of lucid-like dreams recalled by each group. Histogram represents the number of lucid, semi-lucid, and total number of lucid-like dreams reported by the three groups during the experiment. See Table 2 for exact counting. Blue bars represent lucid dreams, brown bars represent semi-lucid dreams, and green bars represent the sum of lucid and semi-lucid dreams for that group.

#### 3.2.2 LucidDreamingVR incorporations in lucid and semi-lucid reports

As VR-Exp participants were asked to provide descriptions of their lucid and semi-lucid dreams, it was possible to qualitatively evaluate whether they included content that may be connected to LucidDreamingVR sessions. Of the 7 lucid dreams of VR-Exp group, 2 included elements of the VR training (see Supplementary materials, reports L3 and L7); of the 19 semi-lucid dreams, one did (see report S1.6). In two of these cases, supposed LucidDreamingVR content incorporation was what triggered lucidity.

#### 3.2.3 Increase in dream recall

Figure 5 shows the average amounts of reported dreams by each group in each week. In it, the label “pre” on the *x*-axis refers to the self-reported number of dreams recalled before the start of the experiment, while the subsequent values represent experimental weeks 1–5. All groups showed an increase in dream recall from baseline to week 1, followed by a steep decrease at Week2 and then a more gradual decline. Across the study, the Control group reported the highest recall, the VR-Control group intermediate recall, and the VR-Exp group had the lowest recall. The proportion of nights with at least one recalled dream was 47.0% in VR-Exp, 52.6% in VR-Control, and 59.5% in Control.

**Figure 5:**
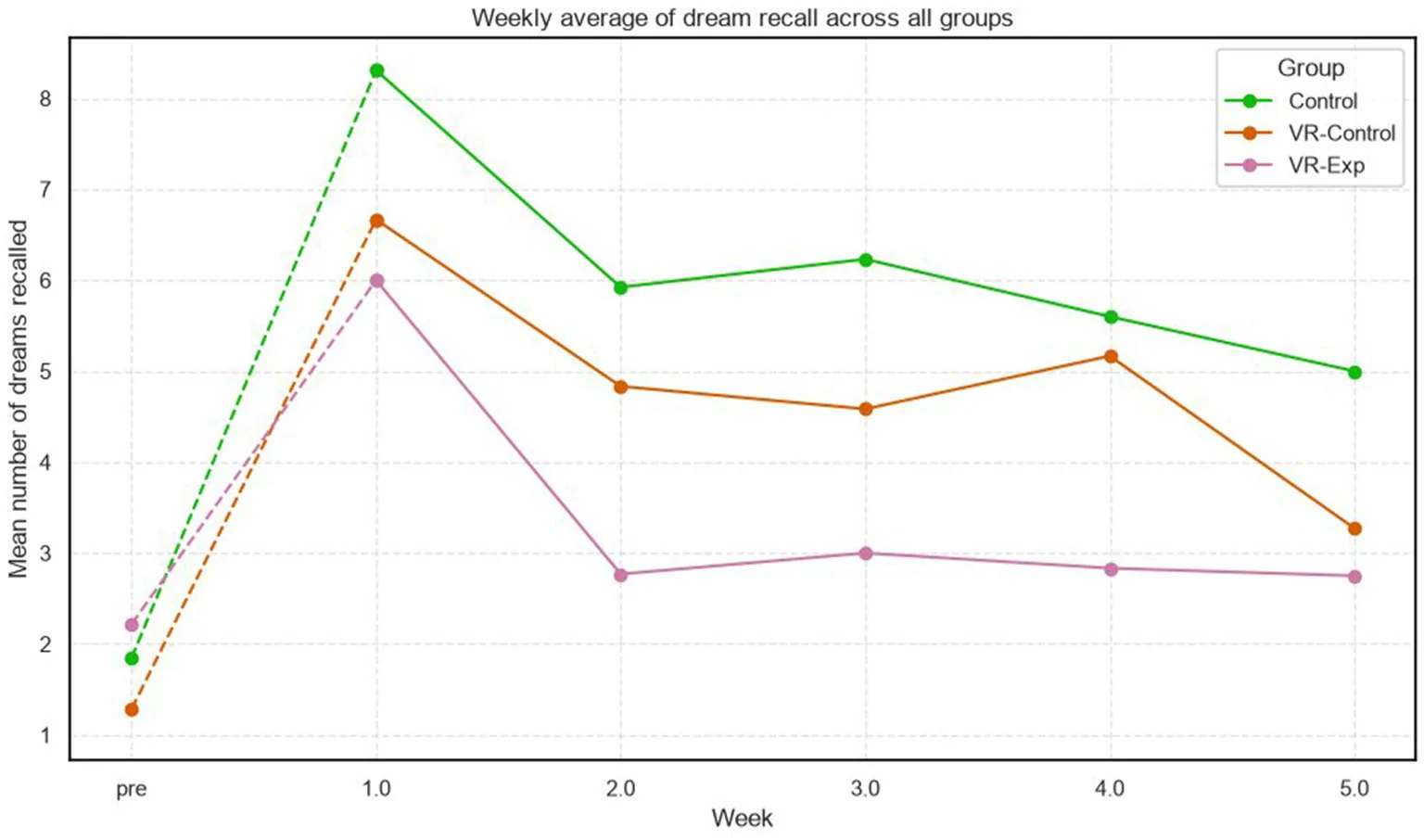
Weekly average of all recalled dreams, irrespective of lucidity classification. Dream recall is visualized here as the average number of dreams reported each week. The label “pre” on the *x*-axis refers to the self-reported number of dreams recalled by participants before the start of the experiment, while the subsequent numbers correspond to the experimental weeks. Green line represents the trend of weekly dream recall in Control group; orange line refers to VR-Control, and the purple line shows dream recall rates for VR-Exp.

### 3.3 LuCiD scale analysis results

#### 3.3.1 Cronbach's α and reliability of LuCiD subscales

Internal consistency was acceptable to high for all LuCiD subscales, with only Dissociation falling below α = 0.80, while remaining acceptable at α = 0.703.

#### 3.3.2 LuCiD differences between lucid, semi-lucid, and ordinary dreams

Differences in LuCiD subscales' average values across nights in which participants reported ordinary, semi-lucid, or lucid dreams are shown in Figure 6. Statistical significance was assessed on subject-level aggregated scores using pairwise Mann–Whitney *U* comparisons with Holm–Bonferroni correction.

**Figure 6:**
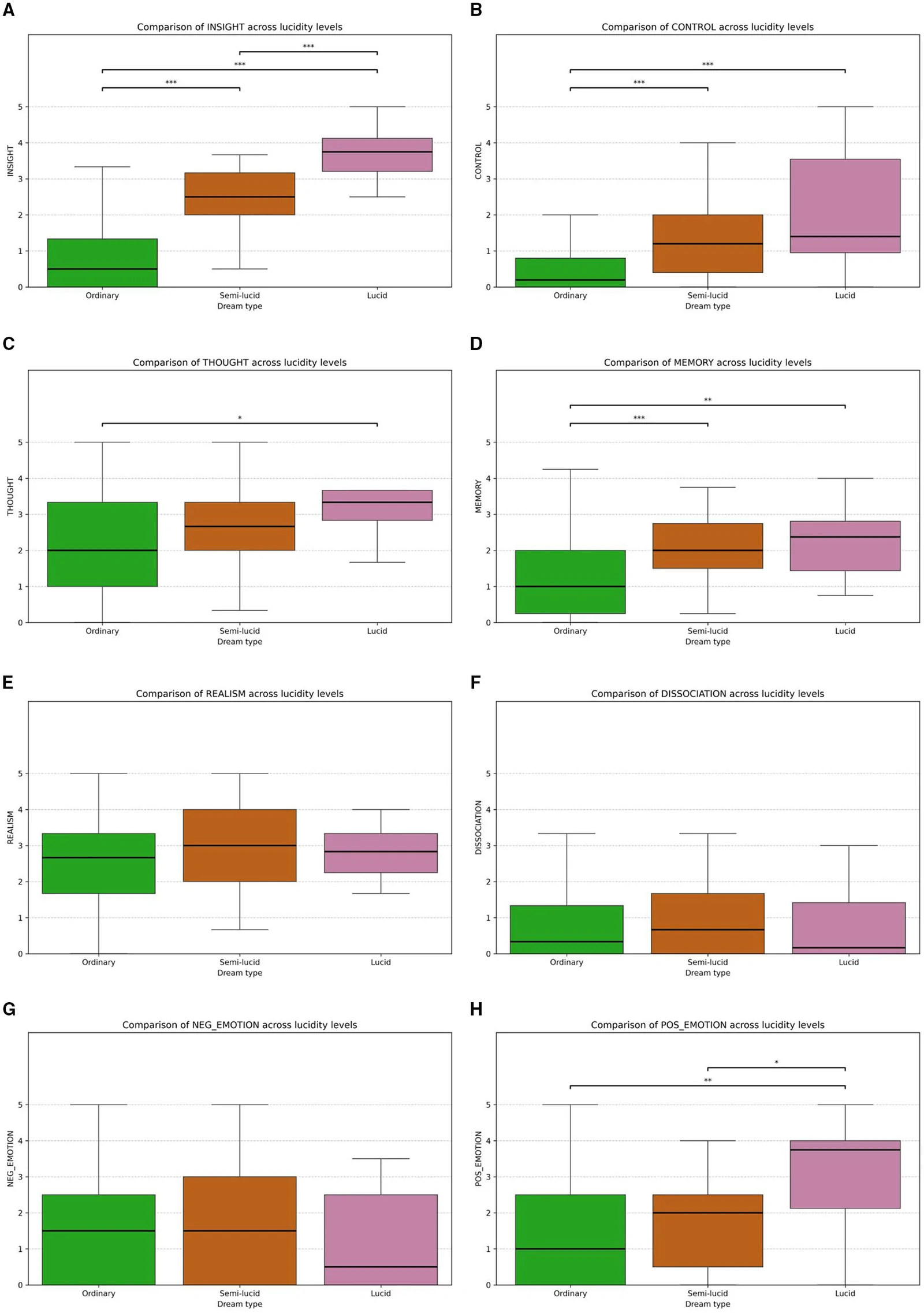
Comparison of all LuCiD subscales across lucidity levels. **(A)** represents LuCiD_insight_ comparison; **(B)** LuCiD_control_; **(C)** LuCiD_thought_; **(D)** LuCiD_memory_; **(E)** LuCiD_realism_, **(F)** LuCiD_dissociation_; **(G)** LuCiD_neg_emotion_; **(H)** LuCiD_pos_emotion_. All values range between 0 and 5. The black lines in the boxplots represent the median and the whiskers represent data distribution outside of the 25th−75th quartile. Here, LuCiD subscales' values were collected from the entire sample, without differentiating by experimental groups. ^*^p < 0.05, ^**^p < 0.01, ^***^p < 0.001.

In this study, fully lucid dreams can be separated from ordinary dreams by having higher values of LuCiD_insight_, LuCiD_control_, LuCiD_thought_, LuCiD_memory_, and LuCiD_pos_emotion_. Semi-lucid dreams are distinguished from ordinary ones by LuCiD_insight_, LuCiD_control_, and LuCiD_memory_. Finally, lucid dreams show higher values of LuCiD_insight_ and LuCiD_pos_emotion_ when confronted with semi-lucid dreams. LuCiD_realism_, LuCiD_dissociation_, and LuCiD_neg_emotion_ were all unable to distinguish between any dream type.

### 3.4 Analysis of consciousness-alteration of VR sessions

As shown in Table 3, within the VR-Control group the PCI subscale Altered State of Awareness decreased between the first and the eighth session, whereas Attention, Direction of Attention, Imagery Amount, and its compound subscale Imagery showed increases over the same interval. No within-subject differences were observed in the VR-Exp group across sessions.

**Table 3:** Statistical comparisons of PCI subscales between groups and sessions.

Variable	Mean 1 ±SD 1	Mean 2 ±SD 2	Test	Statistic	*p*-value (not corrected)	Holm-corrected *p*-value
VR-Exp, Session 1 vs. 8						
–	–	–	–	–	–	–
VR-Control, Session 1 vs. 8						
AS	2.667 ± 1.764	0.967 ± 1.138	Wilcoxon	0.0	0.018	0.406
ATT	2.900 ± 0.527	3.580 ± 0.959	Wilcoxon	2.0	0.043	1.000
DR	1.600 ± 1.255	2.933 ± 1.955	Wilcoxon	0.0	0.012	0.211
IA	0.750 ± 0.950	1.600 ± 1.595	Wilcoxon	0.0	0.016	0.406
IM	1.250 ± 0.882	2.025 ± 1.387	Wilcoxon	4.0	0.028	0.656
VR-Exp vs. VR-Control, Session 1						
AE	3.278 ± 0.948	2.635 ± 1.043	Mann–Whitney *U*	120.5	0.022	0.551
IA	2.615 ± 1.583	1.083 ± 1.379	Mann–Whitney *U*	124.0	0.012	0.323
IM	2.731 ± 1.519	1.458 ± 1.132	Mann–Whitney *U*	119.0	0.027	0.653
RA	4.282 ± 1.201	5.306 ± 0.658	Mann–Whitney *U*	38.0	0.029	0.672
VC	3.333 ± 1.106	4.972 ± 0.881	Mann–Whitney *U*	20.5	0.002	0.050
VR-Exp vs. VR-Control, Session 8						
AS	2.633 ± 1.659	0.967 ± 1.138	Mann–Whitney *U*	77.0	0.043	1.000

The table reports intra-group and inter-group non-parametric comparisons of PCI subscale scores across experimental conditions. For each comparison, the table reports group means and standard deviations, the statistical test used, the corresponding test statistic, the raw p-value and the Holm-corrected p-value. Empty rows indicate that no eligible comparison was available for that condition. Raw p-values should be interpreted descriptively in light of the exploratory nature of the PCI analysis. After Holm–Bonferroni correction, only Volitional Control (VC) remained statistically significant in the between-group comparison at Session 1. AE, Altered Experience; AS, Altered State of Awareness; ATT, Attention; DR, Direction of Attention; IA, Imagery Amount; IM, Imagery (composite of Imagery Amount and Imagery Vividness); RA, Rationality; VC, Volitional Control.

Between-group comparisons indicated that, at session 1, VR-Exp participants reported higher levels of Altered Experience, Imagery Amount and Imagery, while also showing lower scores in Volitional Control and Rationality relative to the VR-Control group. Volitional Control was the only comparison that remained statistically significant after Holm–Bonferroni correction. At session 8, VR-Exp participants exhibited higher Altered State of Awareness scores compared to VR-Control.

## 4 Discussion

This study investigated whether an immersive VR application explicitly designed to rehearse lucid-dream–relevant metacognitive routines (LucidDreamingVR), complete of an OM preparation and embedded within a standard program of dream diary and daily RCs, can enhance lucid-like dreaming as a domain-specific expression of metacognitive state-awareness, relative to baseline training alone and to a content-neutral VR control task (Baird et al., 2019; De Pisapia, 2021; Tormen, 2024).

The principal finding is that participants in the LucidDreamingVR condition reported substantially more lucid-like dreams (lucid + semi-lucid) and a higher proportion of “responders” (participants experiencing at least one lucid-like dream) than either control group. Importantly, this enrichment in lucid-like dreams occurred despite the VR-experimental group reporting fewer ordinary dreams overall, indicating that the effect cannot be reduced to increased dream reporting. Together, these results support the 5value of dream-oriented VR preceded by preparatory OM meditation as a facilitator of lucidity, while also highlighting that VR exposure *per se* is neither sufficient nor uniformly beneficial.

A core challenge in lucid dream induction is transferring waking reflective habits into REM dreaming. Cognitive techniques such as RCs and mnemonic induction aim to increase the probability that a DS triggers a reality-monitoring operation and that this operation is then stabilized long enough to generate insight (“this is a dream”) (Stumbrys et al., 2012). In this context, LucidDreamingVR can be conceptualized as a *rehearsal scaffold* for that transfer problem: it offers repeated, embodied practice in recognizing implausible events and executing reality-check routines within an immersive scenario that approximates key phenomenological properties of dreaming (e.g., violated physical constraints and surreal transitions) (Gott et al., 2021; Kitson et al., 2022). Results of this study also show that interactive VR training content, like general videogames (Gackenbach et al., 2011), can sometimes be incorporated into dreams and may offer crucial cues in recognizing one's dreaming state. This view is consistent with evidence, coming from the only previous controlled study testing VR-assisted lucid dream induction, that VR-enhanced LD training can increase dream lucidity relative to comparison conditions (Gott et al., 2021).

However, such previous study observed a significant benefit of VR only relative to a passive control, while the difference between VR-enhanced and standard lucid-dream training was not significant (Gott et al., 2021). Methodologically, Gott et al. exposed participants to 12 VR sessions based on a range of games selected for their dream-like and surreal qualities, including the custom *Spinoza Café* scenario, while reality checks were practiced as a parallel daytime training component. In the present study, by contrast, LucidDreamingVR was conceived as a more integrated training environment: across eight sessions, dream-sign detection, reality checks, stabilization and lucid-dream–specific actions were progressively rehearsed within the VR experience itself, following a brief open-monitoring meditation. This difference may be relevant because the LD-specific VR condition here showed a higher rate of lucid-like dreams than both standard training and an active VR-control condition, suggesting that the benefit depends not simply on exposure to immersive or bizarre environments, but on embedding dream-relevant metacognitive operations within them. Direct comparison of induction rates should nevertheless remain cautious: Gott et al. (2021) assessed changes in lucidity scores and included polysomnographic eye-signal verification, whereas this research primarily quantified self-reported lucid and semi-lucid dreams across the intervention. The number of recalled dreams per group strengthens the findings: dream recall frequency can correlate with lucid dream recall (Schredl and Erlacher, 2011), and in many induction protocols dream diaries are used partly to increase access to dream content (Schredl, 2002). In the present study, however, both control groups showed higher dream recall rates than the VR-experimental group, while the VR-experimental group nonetheless showed a higher proportion of lucid-like dreams among their reported dreams. This dissociation suggests that the LucidDreamingVR condition enriched the *quality* of dream consciousness, specifically its reflective components, rather than increasing the quantity of remembered dreams. One speculative possibility is that the intervention shifts dream mentation toward more metacognitively marked experiences, which become more salient and thus more likely to be classified and remembered as lucid-like; another possibility is that ordinary dreams without reflective features became less memorable or less likely to be recorded in that group. Distinguishing between these accounts would benefit from additional compliance measures and objective sleep indices.

To complete the picture of dream recall, all three groups showed a marked peak in the number of dreams relative to the retrospective baseline estimate. The subsequent decline is consistent with patterns reported in previous dream diary research (Bernstein and Belicki, 1996; Zadra and Robert, 2012) and may reflect initially high motivation to monitor and record dreams, followed by reduced engagement over time (Zadra and Robert, 2012). However, the present study cannot determine the mechanisms underlying this pattern, as participants' personal dream diaries were not inspected, and diary entries were not compared with daily questionnaire submissions. Motivation was also not assessed over time. Across participants, nights with lucid dreams were characterized by higher LuCiD insight, control, thought, memory, and positive emotion relative to nights with ordinary dreams only, broadly replicating the profile reported in the original LuCiD validation work (Voss et al., 2013). In contrast, dissociation did not differentiate dream types in the present dataset, and its interpretation is constrained by the relatively weaker internal consistency observed for that subscale (Henson, 2001).

In the present study, LuCiD scores were collected at the level of the night rather than of individual dreams. For each item, participants reported the highest value applying to any dream recalled that night. This approach reduced participant burden and allowed potentially relevant features occurring across multiple dreams to be retained, but it also limits the interpretation of the resulting profiles. In a night containing several dreams, high scores on different LuCiD dimensions may originate from different dreams; consequently, the resulting nightly profile does not necessarily characterize a single dream experience. Likewise, a brief episode of lucidity may determine a high LuCiD_insight_ score despite occupying only a small portion of the night's dream experience. This limitation is particularly relevant for novice lucid dreamers, for whom lucidity may be unstable or followed rapidly by awakening. The qualitative reports in the Supplementary materials illustrate this variability: in some cases, participants became lucid and immediately woke up (e.g., report L1), while other reports describe awakening shortly after lucidity emerged (L11; S2.3; S2.4), uncertainty about whether lucidity was “simulated” (S4.2), or loss of lucidity before the dream ended (L4). Thus, the present LuCiD analyses are better interpreted as indexing the maximum expression of lucid-dream-related features within a night, rather than the duration, stability, or overall quality of a single lucid dream.

A further contribution of the present study is the systematic characterization of semi-lucid dreams using the LuCiD framework. Semi-lucid dreams were differentiated from ordinary dreams primarily by increased insight, control and memory, while fully lucid dreams showed higher insight and (relative to semi-lucid) more positive emotional tone. This pattern supports a graded transition model of dream awareness (Mallett et al., 2021; Tyson et al., 1984). The finding that mnemonic activation appears already elevated at the semi-lucid stage suggests that memory access may be an early, and possibly enabling, component in the pathway to lucidity. This aligns with the rationale of mnemonic-based induction strategies such as MILD, which emphasize prospective memory for recognizing the dream state (Stumbrys et al., 2012). While the present data cannot establish temporal precedence, they motivate the hypothesis that memory-related activation may be a necessary but not sufficient condition for full lucidity, with subsequent recruitment of control- and thought-related dimensions contributing to stabilization and elaboration. Such multicomponent characterization may prove useful in refining induction methods and in interpreting partial lucidity reports.

A notable observation is that participants in the VR-control condition reported fewer lucid-like dreams than both the VR-experimental group and the non-VR control group, despite receiving the same number of VR sessions and practicing the same techniques during wake. This pattern is important for cognitive-enhancement research because it suggests that immersive engagement alone was not sufficient; the training environment needed to instantiate the cognitive operations targeted by the intervention. In this case, those operations were anomaly detection, reality monitoring, and reflective state evaluation.

One plausible account is that the control VR task promoted task adaptation and habituation rather than reflective questioning. In line with this, PCI patterns suggested changes over sessions consistent with reduced experiential alteration (decreased PCI altered experience) and increased attentional and imagery-related abilities (increased attention and imagery indices), potentially reflecting familiarity with the environment and reduced novelty (Pekala, 1991). Such adaptation may decrease the salience of “impossible” cues and, consequently, fail to strengthen the coupling between anomaly detection and reality monitoring that RC-based induction relies on (Gott et al., 2021; Stumbrys et al., 2012). Future studies should include structured measures of training credibility/expectancy to clarify whether the VR-control decrement reflects cognitive mechanisms, motivational displacement, or both.

Only participants in the VR-experimental group received an in-headset OM meditation at the beginning of each LucidDreamingVR session. It contained no LD-specific material and participants were not asked to meditate alongside the other at-home practices. Its inclusion in the protocol was motivated by the will to generate a domain-general state of receptive, non-reactive monitoring before the interactive training. Nevertheless, OM meditation may itself facilitate LD by strengthening a non-reactive monitoring stance that allows insight to arise without destabilizing arousal and by enhancing sensitivity to subtle shifts in mental content that could generalize to REM mentation (Wallace, 2010). Because neither control condition included OM meditation, the present study cannot isolate its independent contribution.

Several limitations should be considered when interpreting the present findings. First, all lucid-like outcomes were based on at-home self-report. Although self-report is standard in dream research and was supported here by validated measures of dream consciousness (LuCiD) and established diary methodology (Schredl, 2002; Voss et al., 2013), the gold standard for lucid dream validation remains physiological verification in laboratory or home-polysomnography paradigms with pre-arranged signals (LaBerge et al., 1981). Future studies should incorporate such verification to strengthen inferences about true lucid REM episodes and to reduce potential demand characteristics. Also, the LuCiD scale was employed on a night level and not on the level of individual dreams, as it was designed for.

Second, the sample size was modest although aligned with previous literature (Gott et al., 2021), limiting statistical power for subgroup analyses and for detecting smaller changes in continuous measures such as PCI indices after correction for multiple comparisons. Larger samples would also allow modeling of individual differences that may moderate responsiveness to VR-based training (e.g., baseline dream recall, trait mindfulness, prior meditation, or metacognitive ability) (Gerhardt and Baird, 2024; Schredl and Erlacher, 2011). In particular, prior meditation experience was not assessed; given evidence linking meditation style to lucid dream frequency (Gerhardt and Baird, 2024), controlling for this variable would help distinguish intervention effects from pre-existing tendencies.

Third, the intervention combined multiple components (dream diary, RC, LucidDreamingVR, OM meditation). While this package reflects an ecologically realistic training protocol, it constrains mechanistic attribution. Future work should use factorial or dismantling designs to separate the contributions of dream-relevant VR content, generic VR exposure, and meditation.

A last limitation of the present study is that the semi-lucid category was phenomenologically heterogeneous. Although *post-hoc* inspection indicated that most reports fell within a broader spectrum of lucid-related awareness, ranging from pre-lucid to explicitly lucid experiences, the analyses referred to participants' prospective *maybe* classifications rather than reassigning dreams on the basis of retrospective external coding. This approach preserved the participants' own uncertainty, but it also means that the semi-lucid category should be interpreted as an operational grouping rather than as a phenomenologically uniform state.

In summary, the present study provides preliminary evidence that the LucidDreamingVR approach—combining an OM-based preparation followed by an interactive rehearsal of LD-relevant metacognitive routines, all mediated by VR—can enhance lucid-like dreaming when integrated with standard induction practices. The pattern appears linked to the LD-oriented intervention package rather than to VR exposure alone, is not explained by increased dream recall, and is accompanied by phenomenological patterns consistent with a graded transition from ordinary to semi-lucid to lucid dreaming (Mallett et al., 2021; Tyson et al., 1984; Voss et al., 2013). More broadly, the findings support the use of immersive environments as process-specific cognitive-enhancement tools, particularly when the simulated context is designed to train the cognitive operations required by the target ability. Future work should combine VR-based rehearsal with objective sleep verification, factorial designs, and waking cognitive measures to clarify mechanism, specificity, and transfer.

## Data Availability

The raw data supporting the conclusions of this article will be made available by the authors, without undue reservation.
