# Supplementary material for: Virtual reality as a metacognitive training tool for lucid dreaming
Source: Front Psychol. 2026 Sep 28;17:1924370. doi: 10.3389/fpsyg.2026.1924370 (PMC13654971; doi:10.3389/fpsyg.2026.1924370)
Supplement: Supplementary file 1 [file Supplementary_file_1.pdf]

Virtual Reality as a Metacognitive Training Tool for Lucid Dreaming
Andrea Signorelli, Francesco Tormen, Bruna Dal Bianco, Nicola De Pisapia

Supplementary Materials

1. Reports of Lucid Dreams

The reports are translated from the original Italian with the aim of preserving their wording, experiential structure, and degree of certainty. Names of people and places have been removed or replaced with generic descriptions.

One report was uploaded blank by the dreamer, resulting in 11/12 available reports.

Report L1

I was dreaming that my girlfriend and my friends had come to visit me for a weekend, as had recently happened in waking life. We decided to eat at a delicatessen that served giant pretzels filled with speck. My girlfriend and I each took one, but when we went to the register, the man working there told us that the two pretzels cost 40 euros. At that moment, I realized that I was in a dream and performed a reality check. When the check confirmed that I was actually dreaming, I woke up.

Report L2

It began with a family dinner that I did not want to attend. Then I found myself serving drinks in a bar, and I became lucid while focusing on opening a bottle of wine, because it gave me a sense that something was abnormal. I then covered my eyes with my hands and could still see them too clearly, despite my eyes being closed. I also pinched my nose to check whether I could still breathe. These were the main reality checks I performed in the dream whenever I felt disoriented or noticed that the environment was beginning to fade. The first thing I did was jump out of the window of the cramped bar and fly over a very large and bright square. I then wanted to paint on a canvas in order to send a message, although I do not clearly remember what the message was. To find a canvas, I contacted some friends, who took me to a room with empty walls. Then the alarm woke me up.

Report L3

<u>I entered the kitchen and found objects floating in the air while catching fire</u>. I realized that it might be a lucid dream and, because the room was dark, I tried to turn on the light, but it would not come on. I decided to go back toward the entrance and saw that, instead of the usual door, there was a glass door with light coming through from behind it. I decided to go that way. At a certain point, I found myself interacting with people I know in waking life. I understood that they would never behave that way in reality. On the one hand, I felt that I had control over the dream; on the other, I still felt at the mercy of events. I nevertheless believe that I experienced what a lucid dream is like and that I only need to learn how to "manage" and "control" it.

Authors' comment: this report comes from a VR-Exp participant. The underlined event seems to be closely related to what happens during the first of LucidDreamingVR's sessions in which, once entering the kitchen/dining room area, the user encounters a flaming chair and a floating pizza box. This report was produced the night after the first lab session, suggesting immediate incorporation of LucidDreamingVR's content and successful recognition by the dreamer.

Report L4

I was dreaming something strange. I was inside the world of a game I often play, holding a blue telephone that was shaped like a tube, and I was talking on the phone with a friend. Suddenly, I realized that the situation I was experiencing was not real, but the realization lasted only a very short time and I did not become fully conscious. In other words, I understood that I was in a dream, but it made no difference: I felt disoriented, and within a few seconds I had forgotten it. It was a very superficial awareness. The trigger was the unrealistic scene. I did not perform a reality check in the dream, but I was talking on the phone with someone who was taking part in the same lucid-dream experiment as I was, so perhaps that also helped me notice what was happening.

Authors' comment: this report comes from a VR-Control participant; as a consequence, the underlined event is unrelated to LucidDreamingVR, but shows how videogame content can be incorporated into dreams, as reported by Gackenbach et al. (2011).

Report L5

I would rather not describe last night's lucid dream in detail because it was emotionally very intense and therefore private. At the beginning of the dream, I was not lucid. I do not remember which particular features of the dream made me doubt that I was in waking reality. In any case, after performing a reality check by looking at my hand—it had a strange, impossible shape—I realized that I had become conscious within a dream. I asked to see a person who is important in my life and to be able to speak with them. My request was granted. The meeting was brief, and only a few words were exchanged. I woke up and, in a subsequent dream in which I did not become lucid, I met that person again, just as they had also been present in the previous dream before I became lucid.

Report L6

I found myself inside a museum or gallery whose walls were covered in colored marble and white columns. The structure was enormous, like the interior of a cathedral filled with tall windows through which a great deal of light entered. I approached one of the windows and looked out over an urban landscape, with meadows and rivers farther away. I do not remember exactly what it was—whether a single object or the entire context of the moment—but I sensed that something about the place was strange and that it was unlikely I was really there. I performed a reality check by looking at my hand. It had three widely separated fingers ending in small, gray, conical nails; it looked like a reptile's hand and had webbing between the fingers, like the feet of geese or ducks. I realized that, obviously, it was not my hand and that I was dreaming. I was calm and already knew what I wanted. Suddenly, I was rotated 180 degrees toward a very long, wide, high corridor clad in ochre-colored marble, like a hall of mirrors. At the far end stood a dark bronze portal. I asked to meet a person who is very important to me, the same person I had asked to see in a lucid dream the previous week. As I began to levitate and fly toward the portal, a voice answered that it would not technically be possible for me to meet that person, but that I would only be able to see them. I went through the portal. There was an atrium where people wandered around; I could see them, but perceived them as transparent. I had the distinct impression that they were the people of my unconscious. Beyond the atrium opened an immense white hall packed with people, like a crowded weekend market. I found myself in front of a barrier made of metal bars. Beyond the bars, the person I had asked to see was sitting. Technically, just as the voice had said, I could not reach them because the bars separated us. Since this concerns private and emotionally intense matters, I will not write any more about it here. The lucid dream nevertheless continued, although in some way I began to lose control of it. I was surrounded by a crowd, a procession of people who seemed to be celebrating. They placed me on a sled

at the front of the procession and we moved forward together. There were several famous figures in the crowd, including two well-known actors. One of them approached me with the eccentric expression of one of his film characters. We talked; then I pointed at him, he laughed, and he began to levitate. If I rotated my finger, he rotated in the same direction. It was amusing. We spoke again. I do not clearly remember what he said, but he encouraged me to continue. Perhaps the procession represented life. I do not remember very well what happened next; perhaps we reached the outdoors. In any case, I woke up shortly afterward.

Report L7

In the dream, I was in the center of the town where I live, walking around with a spritz in my hand. At that moment, I thought and said out loud that I was in a dream, and I started performing reality checks; I was indeed in a dream. I then made myself smaller, and when I saw myself from the outside, I was not myself but a non-real character. The environment was not real either; rather, everything was colorful and surrounded by nature. I walked around and, as I met other characters, I changed the color of their clothes as I pleased. I then began flying over this imaginary environment, before waking up shortly afterward. What struck me most was being able to do supernatural things and to transform things according to my will.

Authors' comment: this report comes from a VR-Exp participant; becoming smaller is a "superpower" found in the sixth and seventh LucidDreamingVR sessions, in which the user becomes smaller (by eating a food "thought to be magical" and asking out loud to become smaller, so as to fit in a doll's house or in a passage between rocks). The eight (final) session is set in a very colorful natural place (a magical lake during the night) and its main topic is learning to fly. This report comes from the fifth and final week of training, after having experienced all sessions.

Report L8

The dream was extremely vivid, so much so that at a certain point I began questioning it and realized that it was a dream. I then started moving around the dream environment. I changed the setting a couple of times before returning to the original one, and I performed another reality check when I became uncertain again about whether I was dreaming. I tried to pass a finger through a wall or something similar, but I only ended up breaking jars and walls.

Report L9

I was evaluating someone's artistic work at a school, probably a high school. The assessments seemed to proceed automatically until the final one, when I had to make a choice; that is when I think I became lucid, through the act of choosing something. I did not perform a reality check, but I became aware of the situation. What struck me was not knowing how the dream could continue except through a choice that depended on me.

Report L10

I was in the kitchen and wanted to prepare something new to eat. I picked up a cookbook, looked through the index, chose a recipe, and the reality check consisted of reading that recipe because no instructions appeared. From that point onward, I was certain that I was lucid.

Report L11

I noticed that the sink I was using did not belong in the room I was in, which was the bathroom in my home. I inferred that I was in a dream and tried the reality check of breathing while pinching my nose closed. That made me lucid. Unfortunately, a few moments later the alarm went off and I woke up.

2. Semi-lucid Dream Reports (reported as “maybe” lucid by the dreamers)

The reports are translated from the original Italian with the aim of preserving their wording, experiential structure, and degree of certainty. Names of people and places have been removed or replaced with generic descriptions. The following reports are grouped according to the category recognized by the authors by reading their phenomenological descriptions; False Awakening and Dream Re-entry are retained only as secondary labels.

Four reports were uploaded blank by the dreamers, resulting in 25/29 available reports.

2.1 Lucid

Report S1.1

I fell asleep with the dim bedside lamp on because I was very tired. Then I saw a road and found myself riding a motorcycle at sunset. At a certain point, while I was dreaming of riding, there was also wind, and for some reason I wondered whether the wind moving my hair was actually real, because it did not feel cold in the way it normally would. As I questioned this, I tried to remain as calm as possible and simply experience what was happening without panicking, because I knew that panic would wake me up. I therefore continued riding the motorcycle in the dream and tried to change the temperature of the wind I could feel; then I did the same with the colors of the sky and with the streetlights along the road. It was a beautiful experience. In particular, I found it very pleasant and liberating to be able to control certain aspects, although only basic ones—not the dream plot, just simple things such as colors or temperatures—within a calm and relaxed atmosphere. It was a lovely journey, which I think ended after a few minutes. I woke up to turn off the light, but I did not check the time [as a reality check, T.N.].

Report S1.2

At first, I was not lucid; the dream was unfolding without lucidity. At a certain point, when something I did not want to happen was about to occur, I wondered whether I was dreaming. I became aware that I was dreaming and managed to prevent the unwanted event from happening.

Report S1.3 | *Secondary label: Dream Re-entry*

I was sitting in front of a computer watching a video, but I could see myself from the outside, as though I were observing myself. I was aware that I was in a dream, almost as though it were a lucid dream. The strange thing was that every time I woke up and then fell asleep again, I returned to exactly the same point in the video where I had stopped. It was as though the dream had been paused and then resumed. The entire experience was confusing, like a continuous transition between reality and dreaming, and it left me with a very peculiar feeling. I am fairly sure that I really did wake up because, when I performed reality checks, I was actually in waking reality; however, shortly afterward I would fall asleep again. This happened about three times.

Report S1.4

I was in a seaside house, lying on a bunk bed with my family, when water began coming in through the door and windows. I remembered a previous experience abroad and wondered whether I might be dreaming. I checked the door again and noticed that the water did not come through in the same way each time: sometimes there was a great deal of it, and sometimes very little. I then realized that I was dreaming and calmed down, but I was unable to change anything in the dream and woke up shortly afterward.

Report S1.5 | *Also: False Awakening*

I was not lucid from the beginning, and I do not know whether the experience can be considered lucid because it was a lucid dream within another dream. I was alone in my father's house, and paranormal events began occurring. At a certain point, overwhelmed by fear, I remembered that I was inside a dream and that I could change what was happening according to my will. I therefore transformed the "ghosts" into meatballs. However, within the dream, this episode was itself another dream. In other words, after that moment was over, I "woke up" and described what had happened, but in reality I was still asleep and still dreaming.

Report S1.6

I was at work, arranging candles on a shelf. The candles were not placed directly on the shelf itself but inside a cardboard box with dividers to keep them organized. <u>I turned away for a moment, and when I looked back, they had changed position</u>. I tried turning away and looking back a couple more times and realized that I was dreaming. From that point on, however, it was extremely difficult to keep the dream going. After a very short time (about a minute) I woke up.

Authors' comment: this report comes from a VR-Exp participant; the underlined event is very closely related to a specific dream sign present in LucidDreamingVR, in which a statue and a potted plant would change spatial location whenever the user looks away.

Report S1.7

I was in my room fighting someone, although I do not remember who it was. Then I looked at my hands and they were disproportionate, with one finger much larger than the others. I realized that I was dreaming and experienced a sense of power while fighting hand-to-hand, influencing the events as they unfolded.

Report S1.8

I think I became lucid during the dream. In one particular dream, I was fixing the glass panel of a shower and it broke in my hands, but I was not injured. I thought that I must be dreaming because it was impossible for me not to have been hurt. Then I woke up.

2.2 Prelucid

Report S2.1

Broadly speaking, the dream involved one of my professors as a villain who created an apocalyptic storm that I had to manage by moving my hands and "directing" it to the rhythm of a song playing in the background. Then I wondered what I was doing, and I woke up because of the alarm.

Report S2.2

It began with me standing among a small group of people while inventing a game. I remained consciously involved throughout the process of inventing it. However, I woke up quite abruptly, because of my friend, right in the middle of the conclusion. I therefore did not have time to determine whether the dream was actually lucid.

Report S2.3

I dreamed that I was grocery shopping with a couple of friends, but then they turned into two other friends of mine. At that moment, I wondered whether I might be in a dream, because what had happened was impossible. Then I woke up.

Report S2.4

I dreamed that I was holding a book entitled Ikigai, which a friend had recommended to me in waking life. In the dream, however, the book had a completely different cover, and I wondered whether I was dreaming. Then I woke up.

Report S2.5 | *Also: False Awakening*

In the dream, I saw myself from the outside while I was moving objects around my room. At a certain point, I thought, "This book is not like this; I am in a dream." I then tried to perform reality checks, but I woke up—or at least I thought I had woken up, because I experienced a false awakening. In the dream, I wanted to do certain things but never managed to do them, and I kept waking up and falling asleep again.

Report S2.6

I dreamed that I had cut my own hair and made a mess of it. I remember wondering, during the dream, how I could have cut it from long to shoulder length when, in reality, my hair was already shorter than that. However, the thought was immediately forgotten.

2.3 Partial Lucidity

Report S3.1

I am not really sure whether I had a lucid dream. What I remember is that I was absorbed in working on an anthropology project and discussing it with some classmates. For some reason, it turned into a competition with the others, including a physical one. At a certain point, the professor gave us some advice. I turned around and the scene changed: were we on a boat? I am not sure; it was not very clear. What I do remember is turning back and seeing the sea and some dolphins swimming. I then said out loud, "I want to pet one," and it happened. I think it was a kind of lucid dream because I was very conscious and made a series of deliberate choices, which normally does not happen in my dreams.

Report S3.2

At the end of my dream, I thought in the same way I would have thought in waking life, and I managed not to do what I was supposed to do in the dream.

Report S3.3 | *Also: Dream Re-entry*

I dreamed that I was supposed to meet a friend so that I could take some photographs of him, and we were meant to meet at his house in a nearby town. In the dream, I was already at his house and waited for him together with another friend. The second friend then left, and the first friend messaged me to say that he was running late and asked where we should meet. I replied that I would wait for him at his house, but he called me and said that he no longer lived there. Feeling very anxious, I took the keys and went out

through the door. Meanwhile, there was a man in the house taking a shower. I woke up feeling extremely anxious. During this dream, I woke up and fell asleep again several times, and within the dream I realized that it was not reality because one friend's hair was a different color, because I did not actually know where the other friend lived, and because I wondered why I had the keys to that house. Yes, I performed reality checks when I woke from the dream, and I was indeed awake.

Report S3.4

When the dream reached its crucial point, I felt more aware than usual of what was happening.

Report S3.5

I think I experienced an "instant" of lucidity rather than a fully developed lucid dream. I was witnessing an improbable scene in which people who looked ordinary by present-day standards were dressed in royal garments, perhaps medieval ones. I realized—and it was nothing more than an immediate, ineffable recognition—that this could not be my reality and that what I was observing and experiencing could only be unreal.

Report S3.6

I repeatedly picked up a bottle of correction fluid, then remembered that I could not use it because it was a dream.

Report S3.7

I was in a large gym. There were many people I knew and also some of my pets. A presentation or a speech was taking place, although that is not important. I think that awareness became activated when I started interacting with the people and when I became able to accept or reject the course of events. I am uncertain whether it was a lucid dream because I did not look for confirmation. Instead, I allowed myself to be carried along by the events, since they were positive and pleasant.

Report S3.8

During an argument with an unknown woman, I physically attacked her. When she said that she was going to report me, I replied that we were in a dream. I was not lucid either before or after that moment.

2.4 Uncertainty in lucidity placement

Report S4.1

The dream that I think I had last night involved me and a person who had once been dear to me. Since we were getting along in the dream, I thought, "I must be dreaming". This led me to become aware that I was dreaming, but I do not clearly remember whether I had this thought during the dream itself or while I was in a state between sleep and wakefulness.

Report S4.2

In the dream, I had to speak with police officers in order to solve a case. However, the officers were foreigners, and I needed the help of a female friend who spoke their language so that she could translate the conversations. She was not there, so I decided to call her. While making the call, I thought, "I am having a lucid dream." I performed a couple of reality checks. I pinched my nose, but I could still breathe. I also tried clenching my fist, but I could not feel my fingernails or fingers pressing against the palm of my hand. I then decided to take control of the telephone conversation I was having with my friend. However, I do not remember how the dream continued. It may have ended, or I may have lost lucidity again. I selected the option "maybe" because I am not sure that I was actually conscious. I do not know whether I

dreamed that I was having a lucid dream and was aware of it, or whether I was genuinely aware, for a moment, that I was dreaming.

2.5 Ordinary Dream

Report S5.1

I left the university and found myself with my housemates. We were supposed to go somewhere, but we were late. For some reason, I decided to grab a bus and launch it onto a distant road on a mountain. During the consequent fall, however, the bus plunged off a cliff, and all I said was, "Oops, perhaps I did not calculate the distances very well."

3. Tables of statistical tests

Table S1. Descriptive statistics and between-group comparisons for participants with and without lucid or semi-lucid dreams. No statistically significant differences emerged between the two groups across the examined variables.

Variable	Non Responders Mean (SD)	Responders Mean (SD)	Normality (G0 / G1)	Comparable Variances	Statistical Test	Test Statistic	p-value
Age	24.33 (4.82)	22.30 (2.90)	No / No	Yes	Mann–Whitney U	U = 217.50	.181
Dream Recall	10.13 (2.90)	10.83 (3.26)	Yes / Yes	Yes	Independent-samples t-test	t = −0.668	.508
Lucid Dream Recall	1.00 (1.36)	1.30 (1.72)	No / No	Yes	Mann–Whitney U	U = 172.00	1.000
VR Experience	1.00 (0.93)	0.83 (0.83)	No / No	Yes	Mann–Whitney U	U = 189.00	.612

Table S2. Descriptive lucid-like dream rates and pairwise negative binomial comparisons across experimental groups. Both unadjusted and Holm-adjusted *p* values are reported.

Comparison	Lucid-Like Rate Ratio (95% CI)	Unadjusted p	Holm-adjusted p
Control vs VR-Control	2.07 (0.50–8.65)	.316	.316
VR-Exp vs VR-Control	9.02 (2.36–34.46)	.001	.004
VR-Exp vs Control	4.35 (1.45–13.01)	.009	.017

Table S3. Pairwise Poisson rate-ratio comparisons across experimental groups. The VR-Exp group showed significantly higher lucid-like dream rates than both the VR-Control and Control groups after Holm correction.

Comparison	Rate Ratio (95% CI)	Unadjusted p	Holm-adjusted p
Control vs VR-Control	2.15 (0.68–6.76)	.190	.190
VR-Exp vs VR-Control	8.60 (3.00–24.64)	< .001	< .001
VR-Exp vs Control	4.00 (1.97–8.09)	< .001	< .001

Table S4. Pairwise Fisher's exact tests comparing responder rates across experimental groups. After Holm correction, VR-Exp showed significantly higher responder odds than VR-Control, whereas the other pairwise comparisons were not significant.

Comparison	Responder rate	Odds Ratio (95% CI)	Unadjusted p	Holm-adjusted p
VR-Control vs VR-Exp	33.3% vs 84.6%	0.09 (0.01–0.62)	.015	.046
VR-Control vs Control	33.3% vs 61.5%	0.31 (0.06–1.61)	.238	.475
VR-Exp vs Control	84.6% vs 61.5%	3.44 (0.53–22.43)	.378	.475

Table S5. Cronbach's alpha coefficients for the LuCiD subscales across experimental groups and for the overall sample.

LuCiD subscale	Control	VR-Control	VR-Exp	Overall
Insight	.899	.874	.945	.925
Control	.729	.823	.929	.846
Thought	.888	.882	.919	.902
Realism	.868	.921	.942	.911
Memory	.809	.749	.870	.828
Dissociation	.680	.657	.762	.706
Negative Emotion	.854	.914	.876	.881
Positive Emotion	.922	.938	.942	.934

Table S6. Pairwise Mann–Whitney U comparisons of subject-level aggregated LuCiD subscale scores across ordinary, semi-lucid, and lucid dreams. Holm–Bonferroni-adjusted *p* values are reported for the three pairwise comparisons within each subscale.

LuCiD subscale	Comparison	N1	N2	U	Unadjusted p	Holm-adjusted p	Significance
Insight	Ordinary vs Semi-lucid	38	17	72.5	< .001	< .001	***
Insight	Ordinary vs Lucid	38	10	2.0	< .001	< .001	***
Insight	Semi-lucid vs Lucid	17	10	29.5	.006	.006	**
Control	Ordinary vs Semi-lucid	38	17	299.0	.668	.668	ns
Control	Ordinary vs Lucid	38	10	94.0	.015	.046	*
Control	Semi-lucid vs Lucid	17	10	59.5	.201	.401	ns
Thought	Ordinary vs Semi-lucid	38	17	255.0	.219	.219	ns
Thought	Ordinary vs Lucid	38	10	82.0	.006	.019	*

LuCiD subscale	Comparison	N1	N2	U	Unadjusted p	Holm-adjusted p	Significance
Thought	Semi-lucid vs Lucid	17	10	50.5	.086	.172	ns
Realism	Ordinary vs Semi-lucid	38	17	256.5	.229	.688	ns
Realism	Ordinary vs Lucid	38	10	191.0	.990	.990	ns
Realism	Semi-lucid vs Lucid	17	10	102.0	.406	.813	ns
Memory	Ordinary vs Semi-lucid	38	17	182.0	.010	.021	*
Memory	Ordinary vs Lucid	38	10	64.0	.001	.004	**
Memory	Semi-lucid vs Lucid	17	10	62.0	.258	.258	ns
Dissociation	Ordinary vs Semi-lucid	38	17	344.0	.708	1.000	ns
Dissociation	Ordinary vs Lucid	38	10	235.0	.258	.774	ns
Dissociation	Semi-lucid vs Lucid	17	10	89.5	.833	1.000	ns
Negative Emotion	Ordinary vs Semi-lucid	38	17	370.0	.397	.793	ns
Negative Emotion	Ordinary vs Lucid	38	10	247.0	.151	.454	ns
Negative Emotion	Semi-lucid vs Lucid	17	10	88.5	.878	.878	ns
Positive Emotion	Ordinary vs Semi-lucid	38	17	290.0	.554	.554	ns
Positive Emotion	Ordinary vs Lucid	38	10	63.0	.001	.004	**
Positive Emotion	Semi-lucid vs Lucid	17	10	33.0	.009	.019	*

Table S7. Within-group Wilcoxon signed-rank comparisons of PCI subscale scores between Session 1 and Session 8 in the VR-Exp group. Both unadjusted and Holm-adjusted p-values are reported. No comparisons reached statistical significance after Holm correction.

Variable	Session 1 Mean ± SD	Session 8 Mean ± SD	Test	Statistic	Unadjusted p	Holm-adjusted p
AE	39.200 ± 11.497	34.800 ± 17.656	Wilcoxon	22.5	.643	1.000
BI	7.100 ± 5.587	7.400 ± 5.400	Wilcoxon	21.0	.879	1.000
TS	12.400 ± 4.575	9.000 ± 7.513	Wilcoxon	11.0	.191	1.000
PE	10.600 ± 2.633	10.000 ± 3.682	Wilcoxon	11.5	.703	1.000
MN	9.100 ± 5.043	8.400 ± 7.560	Wilcoxon	17.0	.555	1.000
PA	8.900 ± 5.152	5.800 ± 5.329	Wilcoxon	13.0	.150	1.000
JY	6.200 ± 3.155	5.000 ± 4.497	Wilcoxon	22.0	.611	1.000
SE	0.200 ± 0.422	0.000 ± 0.000	Wilcoxon	0.0	.500	1.000
LO	2.500 ± 3.064	0.800 ± 1.476	Wilcoxon	3.0	.156	1.000
NA	4.700 ± 4.900	2.700 ± 3.889	Wilcoxon	9.0	.438	1.000
AN	1.000 ± 1.886	0.300 ± 0.675	Wilcoxon	0.0	.500	1.000
SD	0.900 ± 1.524	0.400 ± 0.966	Wilcoxon	3.0	.625	1.000

Variable	Session 1 Mean ± SD	Session 8 Mean ± SD	Test	Statistic	Unadjusted p	Holm-adjusted p
FE	2.800 ± 3.360	2.000 ± 3.091	Wilcoxon	15.5	.773	1.000
ATT	14.100 ± 5.195	15.500 ± 4.378	Wilcoxon	17.5	.336	1.000
DR	5.500 ± 4.249	6.400 ± 4.551	Wilcoxon	25.0	.844	1.000
AB	8.600 ± 2.119	9.100 ± 1.853	Wilcoxon	26.0	.951	1.000
IM	10.200 ± 6.070	11.000 ± 5.754	Wilcoxon	23.5	.717	1.000
IA	5.200 ± 3.084	5.600 ± 3.688	Wilcoxon	12.0	.828	1.000
IV	5.000 ± 3.127	5.400 ± 3.273	Wilcoxon	22.0	.602	1.000
SA	13.100 ± 4.408	12.600 ± 4.195	Wilcoxon	13.5	.570	1.000
AS	7.400 ± 4.993	7.900 ± 4.977	Wilcoxon	12.5	.484	1.000
AR	4.400 ± 2.914	4.800 ± 3.553	Wilcoxon	20.0	.805	1.000
RA	13.300 ± 3.860	12.200 ± 4.050	Wilcoxon	17.5	.346	1.000
VC	10.700 ± 3.433	10.700 ± 4.900	Wilcoxon	22.0	.969	1.000
ME	15.100 ± 3.755	15.400 ± 2.836	Wilcoxon	9.5	.906	1.000
ID	7.600 ± 3.806	8.800 ± 3.360	Wilcoxon	6.0	.172	1.000

Table S8. Within-group Wilcoxon signed-rank comparisons of PCI subscale scores between Session 1 and Session 8 in the VR-Control group. Both unadjusted and Holm-adjusted p-values are reported. Although several comparisons reached nominal significance before correction, none remained significant after Holm–Bonferroni adjustment.

Variable	Session 1 Mean ± SD	Session 8 Mean ± SD	Test	Statistic	Unadjusted p	Holm-adjusted p
AE	33.300 ± 14.299	28.200 ± 12.264	Wilcoxon	19.0	.432	1.000
BI	7.500 ± 5.401	5.600 ± 5.929	Wilcoxon	7.0	.313	1.000
TS	10.300 ± 4.900	8.400 ± 6.398	Wilcoxon	16.0	.273	1.000
PE	8.600 ± 2.757	8.600 ± 4.351	Wilcoxon	24.0	.754	1.000
MN	6.900 ± 6.707	5.600 ± 4.789	Wilcoxon	21.0	.533	1.000
PA	6.700 ± 5.478	5.400 ± 5.082	Wilcoxon	16.5	.516	1.000
JY	3.900 ± 2.514	3.500 ± 3.136	Wilcoxon	14.0	.633	1.000
SE	0.800 ± 2.530	0.200 ± 0.632	Wilcoxon	0.0	1.000	1.000
LO	2.000 ± 3.333	1.700 ± 2.359	Wilcoxon	10.5	1.000	1.000
NA	5.600 ± 6.947	3.900 ± 5.626	Wilcoxon	14.0	.633	1.000
AN	0.300 ± 0.675	0.800 ± 1.874	Wilcoxon	2.0	.750	1.000

Variable	Session 1 Mean ± SD	Session 8 Mean ± SD	Test	Statistic	Unadjusted p	Holm-adjusted p
SD	2.600 ± 4.006	1.600 ± 3.134	Wilcoxon	6.5	.469	1.000
FE	2.700 ± 3.302	1.500 ± 2.461	Wilcoxon	8.0	.391	1.000
ATT	14.500 ± 2.635	17.900 ± 4.795	Wilcoxon	1.5	.047	1.000
DR	4.800 ± 3.765	8.800 ± 5.865	Wilcoxon	0.0	.008	.211
AB	9.700 ± 1.889	9.100 ± 3.035	Wilcoxon	8.5	.422	1.000
IM	5.000 ± 3.528	8.100 ± 5.547	Wilcoxon	4.0	.027	.656
IA	1.500 ± 1.900	3.200 ± 3.190	Wilcoxon	0.0	.016	.406
IV	3.500 ± 2.369	4.900 ± 3.213	Wilcoxon	10.5	.352	1.000
SA	14.300 ± 3.401	16.000 ± 2.108	Wilcoxon	7.0	.141	1.000
AS	8.000 ± 5.292	2.900 ± 3.414	Wilcoxon	0.0	.016	.406
AR	4.200 ± 2.898	2.900 ± 2.283	Wilcoxon	12.5	.270	1.000
RA	16.100 ± 2.079	14.800 ± 4.158	Wilcoxon	11.0	.359	1.000
VC	15.100 ± 2.726	13.400 ± 4.326	Wilcoxon	9.5	.273	1.000
ME	15.200 ± 3.155	16.700 ± 2.214	Wilcoxon	2.0	.250	1.000

Variable	Session 1 Mean ± SD	Session 8 Mean ± SD	Test	Statistic	Unadjusted p	Holm-adjusted p
ID	7.500 ± 4.478	6.900 ± 4.771	Wilcoxon	21.0	.910	1.000

Table S9. Between-group Mann–Whitney U comparisons of PCI subscale scores between the VR-Exp and VR-Control groups at Session 1. Both unadjusted and Holm-adjusted p-values are reported. After Holm–Bonferroni correction, only Volitional Control (VC) remained statistically significant.

Variable	VR-Exp Mean ± SD	VR-Control Mean ± SD	Test	Statistic	Unadjusted p	Holm-adjusted p
AE	42.615 ± 12.319	34.250 ± 13.559	Mann–Whitney U	120.5	.022	.551
BI	8.000 ± 5.244	7.583 ± 4.963	Mann–Whitney U	84.0	.764	1.000
TS	13.077 ± 4.271	10.667 ± 4.519	Mann–Whitney U	114.0	.052	1.000
PE	11.538 ± 3.152	8.833 ± 2.691	Mann–Whitney U	114.0	.051	1.000
MN	10.000 ± 4.690	7.167 ± 6.279	Mann–Whitney U	114.0	.052	1.000
PA	8.538 ± 4.521	7.917 ± 6.259	Mann–Whitney U	87.0	.642	1.000
JY	6.308 ± 2.750	4.500 ± 3.060	Mann–Whitney U	107.0	.119	1.000
SE	0.308 ± 0.630	0.667 ± 2.309	Mann–Whitney U	88.0	.418	1.000
LO	1.923 ± 2.871	2.750 ± 3.646	Mann–Whitney U	61.0	.341	1.000

Variable	VR-Exp Mean ± SD	VR-Control Mean ± SD	Test	Statistic	Unadjusted p	Holm-adjusted p
NA	3.923 ± 4.591	5.250 ± 6.426	Mann–Whitney U	70.5	.696	1.000
AN	0.769 ± 1.691	0.250 ± 0.622	Mann–Whitney U	84.5	.640	1.000
SD	0.769 ± 1.363	2.500 ± 3.729	Mann–Whitney U	58.5	.243	1.000
FE	2.385 ± 3.097	2.500 ± 3.030	Mann–Whitney U	73.5	.821	1.000
ATT	14.692 ± 4.679	15.333 ± 3.420	Mann–Whitney U	73.0	.806	1.000
DR	6.077 ± 4.112	5.500 ± 3.989	Mann–Whitney U	83.0	.806	1.000
AB	8.615 ± 2.063	9.833 ± 1.749	Mann–Whitney U	49.0	.113	1.000
IM	10.923 ± 6.075	5.833 ± 4.529	Mann–Whitney U	119.0	.027	.653
IA	5.231 ± 3.166	2.167 ± 2.758	Mann–Whitney U	124.0	.012	.323
IV	5.692 ± 3.199	3.667 ± 2.425	Mann–Whitney U	105.5	.136	1.000
SA	12.615 ± 4.312	14.167 ± 3.099	Mann–Whitney U	63.0	.428	1.000
AS	8.615 ± 5.268	8.250 ± 5.190	Mann–Whitney U	77.5	1.000	1.000
AR	4.000 ± 2.708	4.000 ± 2.697	Mann–Whitney U	76.0	.934	1.000
RA	12.846 ± 3.602	15.917 ± 1.975	Mann–Whitney U	38.0	.029	.672

**Variable**	**VR-Exp Mean ± SD**	**VR-Control Mean ± SD**	**Test**	**Statistic**	**Unadjusted p**	**Holm-adjusted p**
VC	10.000 ± 3.317	14.917 ± 2.644	Mann–Whitney U	20.5	.002	.050
ME	14.538 ± 4.371	15.417 ± 2.968	Mann–Whitney U	72.5	.779	1.000
ID	7.846 ± 3.460	7.167 ± 4.549	Mann–Whitney U	79.0	.978	1.000

Table S10. Between-group Mann–Whitney U comparisons of PCI subscale scores between the VR-Exp and VR-Control groups at Session 8. Both unadjusted and Holm-adjusted p-values are reported. Although Altered State of Awareness (AS) reached nominal significance before correction, no comparison remained statistically significant after Holm–Bonferroni adjustment.

Variable	VR-Exp Mean ± SD	VR-Control Mean ± SD	Test	Statistic	Unadjusted p	Holm-adjusted p
AE	34.800 ± 17.656	28.200 ± 12.264	Mann–Whitney U	61.5	.405	1.000
BI	7.400 ± 5.400	5.600 ± 5.929	Mann–Whitney U	60.0	.469	1.000
TS	9.000 ± 7.513	8.400 ± 6.398	Mann–Whitney U	49.5	1.000	1.000
PE	10.000 ± 3.682	8.600 ± 4.351	Mann–Whitney U	55.5	.704	1.000
MN	8.400 ± 7.560	5.600 ± 4.789	Mann–Whitney U	58.5	.542	1.000
PA	5.800 ± 5.329	5.400 ± 5.082	Mann–Whitney U	51.5	.939	1.000
JY	5.000 ± 4.497	3.500 ± 3.136	Mann–Whitney U	60.5	.445	1.000

Variable	VR-Exp Mean ± SD	VR-Control Mean ± SD	Test	Statistic	Unadjusted p	Holm-adjusted p
SE	0.000 ± 0.000	0.200 ± 0.632	Mann–Whitney U	45.0	.368	1.000
LO	0.800 ± 1.476	1.700 ± 2.359	Mann–Whitney U	42.0	.505	1.000
NA	2.700 ± 3.889	3.900 ± 5.626	Mann–Whitney U	40.0	.442	1.000
AN	0.300 ± 0.675	0.800 ± 1.874	Mann–Whitney U	45.0	.654	1.000
SD	0.400 ± 0.966	1.600 ± 3.134	Mann–Whitney U	43.5	.551	1.000
FE	2.000 ± 3.091	1.500 ± 2.461	Mann–Whitney U	51.5	.932	1.000
ATT	15.500 ± 4.378	17.900 ± 4.795	Mann–Whitney U	33.5	.224	1.000
DR	6.400 ± 4.551	8.800 ± 5.865	Mann–Whitney U	36.0	.304	1.000
AB	9.100 ± 1.853	9.100 ± 3.035	Mann–Whitney U	45.5	.760	1.000
IM	11.000 ± 5.754	8.100 ± 5.547	Mann–Whitney U	68.0	.185	1.000
IA	5.600 ± 3.688	3.200 ± 3.190	Mann–Whitney U	67.5	.193	1.000
IV	5.400 ± 3.273	4.900 ± 3.213	Mann–Whitney U	57.0	.621	1.000
SA	12.600 ± 4.195	16.000 ± 2.108	Mann–Whitney U	24.5	.055	1.000
AS	7.900 ± 4.977	2.900 ± 3.414	Mann–Whitney U	77.0	.043	1.000

Variable	VR-Exp Mean ± SD	VR-Control Mean ± SD	Test	Statistic	Unadjusted p	Holm-adjusted p
AR	4.800 ± 3.553	2.900 ± 2.283	Mann–Whitney U	67.0	.209	1.000
RA	12.200 ± 4.050	14.800 ± 4.158	Mann–Whitney U	26.5	.080	1.000
VC	10.700 ± 4.900	13.400 ± 4.326	Mann–Whitney U	31.0	.159	1.000
ME	15.400 ± 2.836	16.700 ± 2.214	Mann–Whitney U	36.5	.292	1.000
ID	8.800 ± 3.360	6.900 ± 4.771	Mann–Whitney U	58.0	.567	1.000
